# Maternal influenza vaccination during pregnancy and the risk of adverse pregnancy and birth outcomes

**DOI:** 10.3389/fphar.2025.1691111

**Published:** 2026-01-05

**Authors:** Vanina Tchuente, Odile Sheehy, Gina Muckle, Mark Walker, William D. Fraser, Anick Bérard

**Affiliations:** 1 Research Center, CHU Ste-Justine, Montreal, QC, Canada; 2 Université Laval, Québec, QC, Canada; 3 University of Ottawa, Ottawa, ON, Canada; 4 Centre de Recherche du Centre Hospitalier de l’Université de Sherbrooke, Montréal, QC, Canada; 5 Faculty of Medicine, Université Claude Bernard Lyon 1, Lyon, France; 6 Faculty of Pharmacy, University of Montreal, Montreal, QC, Canada

**Keywords:** influenza vaccine, low birthweight, maternal influenza, pregnancy, preterm birth, small for gestational age, spontaneous abortion

## Abstract

**Background:**

Influenza vaccination is recommended during pregnancy. We aimed to assess pregnancy and newborns’ outcomes.

**Methods:**

Participants were recruited in their first trimester of gestation, from 05/25/2010 to 08/30/2012. Data were collected, each trimester through telephone-administered interviews. Influenza vaccine exposure was defined as a report of AH1N1 or seasonal influenza vaccine, within 6 months before the last menstrual period or during pregnancy. To answer our different objectives, three sub-cohorts were created: a case-control study to assess the risk of spontaneous abortion (SA), a sub-cohort to assess the risk of prenatal maternal influenza, and a birth cohort to assess the risk of preterm birth (PTB), low birth weight (LBW), small for gestational age (SGA), and infant hospitalization. Multivariate logistic regressions were used to quantify these associations.

**Results:**

In the case-control study (n = 418), after adjustment, no significant association was found between influenza vaccination and the risk of SA (aOR: 0.53, 95% CI: 0.10–2.68). In the sub-cohort of 2,114, 10.0% were exposed to influenza vaccine. Maternal influenza-like symptoms prevalence was 26.5% in the vaccinated group and 25.8% in the unvaccinated group. No significant association was found between the risk of prenatal influenza and influenza vaccine (aOR: 0.92, 95%CI: 0.66–1.29). In the birth cohort of 2,046, 13.9% newborns were exposed *in-utero* to influenza vaccine. No significant association was found between influenza vaccination and the risk of PTB (aOR: 1.30, 95%CI 0.77–2.19), SGA (aOR: 1.13, 95%CI 0.71–1.79), LBW (aOR: 0.94, 95%CI 0.31–2.26), all-cause hospitalization (aOR: 1.02, 95%CI 0.65–1.61).

**Conclusion:**

Influenza immunization during pregnancy does not seem to be associated to pregnancy and birth adverse effects.

## Introduction

During pregnancy, individuals experience physiological changes in their cardiopulmonary and immunological systems (Racicot et al., 2014). Thus, pregnancy increases susceptibility to infectious diseases and respiratory pathogens such as influenza (Rasmussen et al., 2012). Influenza vaccination has been administered to pregnant individuals in the United States for over six decades, following early evidence that influenza infection during pregnancy was associated with adverse maternal and fetal outcomes (Munoz, 2012). Several studies investigating vaccination during pregnancy have demonstrated a protective effect against influenza illness (Pierce et al., 2011; Moro et al., 2011; Munoz, 2012). Nonetheless, many of these studies were constrained by limited statistical power due to small sample sizes (Munoz, 2012). Since 2004, the Centers for Disease Control and Prevention and the American College of Obstetricians and Gynecologists have recommended influenza vaccination for all pregnant individuals as a routine recommendation. In Canada, prior to 2007, influenza vaccination during pregnancy was encouraged, particularly among individuals at increased risk of influenza-related complications (e.g., those with underlying comorbidities (Public Health Agency of Canada, 2023)). However, since 2007, the National Advisory Committee on Immunization (NACI) recommends annual seasonal non-live influenza vaccination in pregnant individuals, and in children aged 6 months and older (Immunization and N.A.C.O., 2018). The uptake of vaccination during pregnancy remains low, which can be explained by the lack of direct recommendation of professional healthcare, the limited access of vaccines during prenatal visit, the lack of education of the safety of vaccination, raising concerns in the pregnant population (Cavaliere et al., 2021; Vilca et al., 2021; Abu-Raya et al., 2020; Vilca et al., 2020).

Evidence showed that influenza in pregnancy was associated with increased risk of complications such as hospitalizations or pneumonia, pregnancy loss, low birth weight (LBW) or preterm birth (PTB). In fact, during the 2009 H1N1 pandemic, pregnant individuals with severe influenza illness had greater risk of preterm birth (PTB) (RR 2.39, 95%CI: 1.64–3.49 (Doyle et al., 2013) and RR: 4.0, 95%CI: 2.71–5.90 (Pierce et al., 2011)). They also had a significantly increased risk of fetal death following maternal H1N1pdm09 influenza disease (RR 1.91, 95%CI: 1.07–3.41 for mild-to-moderate disease (Håberg et al., 2013) and 4.2, 95% CI: 1.42–12.4 for severe disease (Pierce et al., 2011; Fell et al., 2017a)). Currently, leading health authorities—including the American College of Obstetricians and Gynecologists (ACOG), NACI, and the World Health Organization (WHO)—recommend influenza vaccination during pregnancy, citing that the benefits of immunization outweigh potential risks. Indeed, influenza illness poses a greater threat to both pregnant individuals and their infants than the vaccine itself. Despite these recommendations, several studies have highlighted potential biases in the existing literature and have called for improved research methodologies to independently assess the safety profile of influenza vaccination during pregnancy.

Moreover, studies have shown the effectiveness of the influenza vaccination during pregnancy on both pregnant individuals and infants less than 6 months old (Getahun et al., 2019; Nunes and Madhi, 2018; Thompson et al., 2019). In their study, Gatehun et al observed that the influenza vaccination was associated with a decreased risk of maternal influenza (OR: 0.49; 0.39–0.62) (Getahun et al., 2019). However, in 2017, Donahue et al. (2017) in their study found that the influenza vaccine in the preceding 28 days over two seasons (2010-11, 2011-12) was associated with an increased risk of spontaneous abortion (SA). Nonetheless, a 2019 study by the same authors found no association between SA and influenza vaccination during the 2012-13, 2013-14 and 2014–15 seasons (Donahue et al., 2019).

Additionally, some evidence suggests that maternal influenza immunization may reduce adverse birth outcomes (Steinhoff et al., 2014). Some studies reported a significant risk reduction for PTB (from 13% to 29%) (Giles et al., 2019; Rolfes et al., 2019), low birthweight (LBW) (from 18% to 26%) (Giles et al., 2019; Nunes et al., 2016) while others reported no significant association between prenatal influenza vaccine and PTB (Fell et al., 2015; Jeong et al., 2019; Omer et al., 2020), LBW (Jeong et al., 2019; Omer et al., 2020) and small for gestational age (SGA) (Giles et al., 2019; Rolfes et al., 2019; Nunes et al., 2016; Jeong et al., 2019; Omer et al., 2020).

Despite the available studies, the evidence regarding the impact of maternal influenza vaccination on pregnancy and birth outcomes seems inconsistent, particularly in terms of the influenza vaccine and birth outcomes such as PTB, LBW. Given these inconsistencies and using a primary research database completed within Canada, we aimed to (1) assess the risk of SA following influenza vaccine during pregnancy, (2) evaluate the association between influenza vaccine and maternal influenza during pregnancy, and (3) determine the association between *in-utero* exposure to influenza vaccine and the risk of PTB, LBW, SGA, and hospitalizations.

## Methods

### Study population

This study was conducted within the 3D Cohort Study. Details of the 3D Cohort Study have been described elsewhere (Fraser et al., 2016). Briefly, the 3D study recruited 2,366 individuals and their partners at one of nine study centers. The recruitment was done during the first-trimester prenatal visits (8–14 weeks) at the study hospitals and infertility clinics, between May 25, 2010 and August 30, 2012. Individuals were eligible to be part of this cohort if they: (a) were between 18 and 47 years old at the time of recruitment, and (b) were able to read and understand French or English. Individuals were excluded if they (a) were current users of intravenous drugs, (b) had severe illnesses or life-threatening conditions, (c) had multiple gestation pregnancies. While the study follow-up was conducted by the research nurses, infant follow-up was conducted by a team of nurses and research assistants trained in developmental psychometrics. After the first visit during the first trimester, individuals were seen twice during pregnancy (at 20–24 weeks and 32–35 weeks of pregnancy), and at delivery. *Postartum* follow-ups were at 3 months, 1 year, and 2 years after birth.

### Data collection

Each trimester a telephone interviewer-administered questionnaire was used to collect exposures of interest, including maternal characteristics such as individual’s date of birth, gestational age, ethnicity, marital status, socio-economic status (annual income, education, occupation), weight, height. Moreover, pregnancy and medical history, family medical history, vaccination status, maternal comorbidities, and influenza during pregnancy were also collected in the questionnaire. Information on maternal lifestyle during pregnancy (smoking, alcohol consumption, illicit drug use) was also collected. At delivery, information on the newborn, such as birth status, date of birth, sex, and weight, was collected. Furthermore, at the 3-month postpartum interview, data on the baby’s health and hospitalizations were collected.

### Study design

Depending on the study objectives, we built three sub-cohorts: one case-control to assess the risk of SA following influenza vaccine during pregnancy, a retrospective cohorts of pregnant persons to evaluate the association between influenza vaccine and maternal influenza during pregnancy, a retrospective birth cohort to determine the association between *in-utero* exposure to influenza vaccine and the risk of PTB, LBW, SGA, and hospitalizations.

### Exposure

We used three different definitions of the influenza vaccination depending on the study objective.

To assess the risk of SA, pregnant individuals who reported an A (H1N1) or seasonal influenza vaccine in the questionnaire and had their vaccination before the reference date (date of SA for cases or corresponding end of follow-up for controls) were considered exposed. Those who reported receiving an influenza vaccine but were vaccinated after the reference date, or did not report any vaccination, were considered unexposed (Supplementary Figure S1a).

To determine the risk of maternal influenza during pregnancy, individuals who reported receiving an A (H1N1) or seasonal influenza vaccine in the questionnaire and had their last vaccination within 6 months before the last menstrual period (LMP) date were considered exposed. Those who did not report any vaccination or had their last vaccination more than 6 months prior LMP date or during pregnancy were considered unexposed (Supplementary Figure S1b). This classification was chosen because the date of the illness was not available, making it impossible to determine whether illness occurred before or after vaccination in individuals vaccinated during pregnancy (Supplementary Figure S1b). Moreover, we selected the threshold of 6 months, since flu vaccination lasts at least 6 months in average for a person.

To evaluate the risk of neonatal outcomes (preterm birth, LBW, SGA and infant’s hospitalizations within the first 3 months of life), *in utero* exposure to influenza vaccination was defined as being exposed to an A (H1N1) or seasonal influenza vaccine if individuals reported receiving the vaccine in the questionnaire at any time during pregnancy or within 6 months prior LMP date, provided that the vaccine season year matched the season of pregnancy completion (Supplementary Figure S1c).

### Outcomes

#### Spontaneous abortion

Cases of SA were defined as any report of SA during the follow-up interviewer-administered questionnaire at the second visit (between 20 and 24 weeks of pregnancy). Gestational age was also reported through the questionnaire. The estimated date of SA was calculated by adding the reported gestational age at the time of SA to the date of LMP; or by adding the gestational age of 20 weeks to the LMP date, when there was no information on gestational age. We randomly matched potential controls to cases using a 10:1 ratio. For each case, ten controls were selected to increase the statistical power and the precision of the estimated associations, given the limited number of cases (Katki et al., 2023). First, they were matched on the LMP case’s dates, within 15 days. This criterion was chosen to control for the season of the pregnancy, as vaccination schedules are seasonal, ensuring both cases and controls were likely exposed to a similar seasonal condition during pregnancy. In addition, cases and controls were matched on the conception season year. Lastly, cases and controls were matched on pregnancy duration. Given that we matched on those variables, and to avoid overmatching, maternal age was considered in the multivariate analysis allowing us to account for its confounding effect (Pearce, 2016). Ultimately, the reference date for each case-control pair was defined as the SA date for the case and end of follow-up (LMP + pregnancy duration) for the control.

We excluded elective abortions, molar pregnancies, and therapeutic terminations.

#### Maternal influenza during pregnancy

Data on maternal influenza-like symptoms during pregnancy were collected through questionnaires. On the first visit, individuals were asked if they had colds and flu-like symptoms. They were considered to have had influenza-like symptoms during the first trimester if they reported these symptoms.

#### Birth outcomes

Data on newborns were collected through questionnaires at delivery and at 3-months postpartum visits such as preterm birth (delivery at <37 weeks), small for gestational age (<10th percentile), and low birthweight (weight <2,500 g), hospitalization as well as birth date, birth weight, sex, health status (lower and upper tract infections).

### Covariates

Potential confounders were considered in our different multivariate analyses. We included maternal characteristics (maternal age, postsecondary education, marital status (living alone, or not), household annual income (<CAD40,000; 40,000–80,000; ≥80,000), born in Canada, ethnicity). Moreover, maternal parity, first-visit BMI, smoking status, alcohol intake, illicit drug use, ART) were also considered in the adjustment. In addition, maternal comorbidities before or during pregnancy (diabetes, asthma, thyroid disease, anemia, hypercholesterolemia, hypertension, hepatitis, gastrointestinal disease; sexually transmitted disease, depression)) and flu-risk exposure (defined as the number of days an individual was exposed to the influenza season (October–April) from LMP to the end of pregnancy, regardless of the outcome) were accounted in the multivariate analyses. We created this variable to determine the number of days an individual was potentially at risk of having an influenza vaccination. All these characteristics were reported in the questionnaire during the interviews. Missing values for some covariates were replaced by random imputation using the distribution of the variable among patients with complete data.

### Statistical analysis

To address our various objectives, we performed multiple descriptive analyses. First, we compared cases of SA to controls according to their vaccine status and covariates status. Second, we compared maternal influenza-like symptoms status during first trimester of pregnancy and covariates based on influenza vaccine status before pregnancy. Finally, we compared neonatal characteristics based on *in utero* exposure to the influenza vaccine. For all our descriptive analyses, we used t-test for continuous variables and χ2 test for categorical variables (or Fisher exact test when category sample sizes were <5) to determine statistical differences between the exposed and unexposed groups.

Based on our objectives, we conducted several regression analyses. First, we conducted a multivariate conditional logistic regression to assess the risk of SA following influenza vaccination. Second, we conducted a multivariate logistic regression to quantify the association between influenza vaccination before pregnancy and maternal influenza-like symptoms during first trimester. Finally, we conducted a multivariate logistic regression to assess the association between *in utero* exposure to influenza vaccine and the risk of preterm birth, LBW, SGA and hospitalizations during the first 3 months of life.

### Sensitivity analysis

We performed two sensitivity analyses. First, since the flu vaccine is administered seasonally, we selected cases of SA that occurred only during vaccine season, from October 1st to April 1st. Potential controls had the same inclusion criteria (except for the SA) and were randomly matched in a 10:1 ratio with LMP dates within 15 days of the case’s LMP and matched on conception season year and duration. Finally, the reference date for each case-control pair was defined as the SA date for the case and end of follow-up (LMP + pregnancy duration) for the control. Furthermore, we conducted a multivariate logistic regression to assess the association between newborn outcomes and *in utero* exposure to the flu vaccine, stratified by the timing of vaccine shot (6 months before LMP, during 1^st^ trimester, during 2^nd^ trimester and during 3^rd^ trimester; all groups mutually exclusive).

## Results

### Spontaneous abortion and influenza vaccination exposure

**FIGURE 1:**
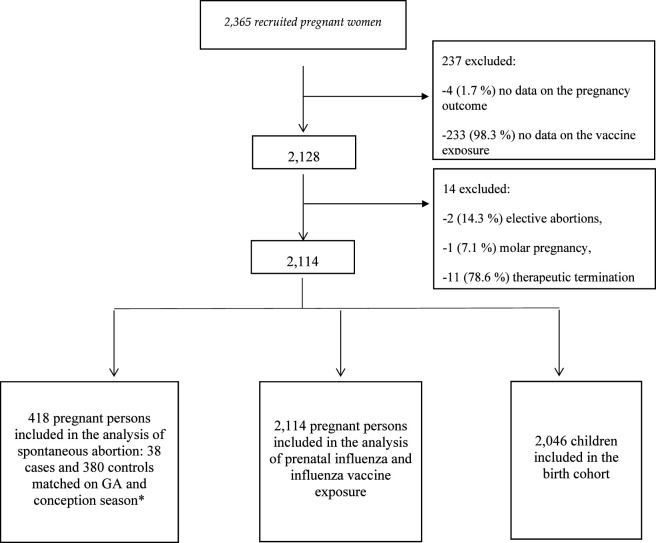
Flow chart describing the selection of our sub-cohorts. *Season year of vaccine period (from October 1st, 2010 to September 30th, 2011, season year is 2011). GA, gestational age.

In the case-control assessing the risk of SA, 418 pregnant individuals were included, consisting of 38 cases and 380 matched controls (Figure 1). The characteristics of individuals with SA and those without SA were similar. The prevalence of the influenza vaccination was lower in the cases (5.3%) compared to the controls (7.6%) (Table 1). After adjusting for potential confounders, we found no significant difference between the influenza vaccine exposure and the SA (adjusted odds ratio (aOR): 0.53, 95% confidence interval (CI): 0.10–2.68) (Table 2).

**TABLE 1:** Maternal characteristics of spontaneous abortion cases and controls.

Characteristics	Cases n = 38 (%)	Controls n = 380 (%)	*p*
Influenza vaccine exposure	​	​	0.5952
Yes	2 (5.3)	29 (7.6)	​
No	36 (94.7)	351 (92.4)	​
Maternal age at reference date^a^, years, mean ± SD	33.3 ± 5.5	31.5 ± 4.7	0.0569
Gestational age at reference date^a^, weeks, mean ± SD	16.0 ± 4.3	16.0 ± 4.2	1.0000
Post-secondary education	31 (83.8)	347 (91.6)	0.1314
Missing	1	1	
Household annual income, CAD	​	​	0.1011
<40,000	4 (10.5)	67 (18.2)	​
40,000–80,000	19 (50.0)	122 (33.2)	​
≥80,000	15 (39.5)	179 (48.6)	​
Missing	​	12	​
Marital status: Living alone	2 (5.3)	24 (6.3)	1.0000
Born in Canada	27 (71.173.0)	278 (73.4)	0.9604
Missing	1	1	
Maternal ethnicity - Caucasian^b^	34 (89.5)	321 (84.5)	0.4114
Parity	​	​	0.1679
0	15 (39.5)	205 (54.0)	​
1	17 (44.7)	116 (30.5)	​
≥2	6 (15.8)	59 (15.5)	​
Body Mass Index^c^, (kg/m^2^), mean ± SD	24.3 ± 5.1	25.1 ± 5.4	0.4327
Missing	3	15	
Maternal lifestyle during pregnancy			
Smoking	8 (21.1)	48 (12.6)	0.1462
Coffee intake	20 (52.6)	253 (66.68)	0.0809
Missing		1	
Illicit drug	2 (5.3)	13 (3.4)	0.6367
Use of assisted reproductive technologies	4 (10.5)	48 (12.6)	1.0000
Season year at conception date^d^	​	​	1.0000
2010	5 (13.1)	50 (13.1)	​
2011	21 (55.3)	210 (55.3)	​
2012	12 (31.6)	120 (31.6)	​
Maternal comorbidities during pregnancy			
Diabetes	0 (0.0)	0 (0.0)	-
Asthma	6 (15.8)	37 (9.7)	0.2590
Thyroid disease	0 (0.0)	24 (6.3)	0.1504
Missing	​	1	​
Anemia	0 (0.0)	18 (4.79)	0.3859
Missing	3	10	
Hypercholesterolemia	0 (0.0)	1 (0.3)	1.0000
Missing		3	
Hypertension	2 (5.3)	1 (0.3)	0.0229
Missing		1	
Hepatitis	1 (2.6)	1 (0.3)	0.1741
Missing		1	
Gastro-intestinal disease	3 (7.9)	21 (5.56)	0.4633
Missing	1	4	
Sexually transmitted infection^e^	0 (0.0)	26 (6.8)	0.1525
Depression	1 (2.6)	5 (1.3)	0.4409
Missing		4	
Maternal influenza during pregnancy	7 (18.4)	91 (24.0)	0.4433
Flu-risk exposure^f^, days, mean ± SD	79.2 ± 49.5	71.5 ± 43.5	0.3050

SD, standard deviation; CAD, Canadian dollars.

^a^ The SA date for the case and end of follow-up (LMP + pregnancy duration) of the control.

^b^ Caucasian = White, East-Asian, South-Asian, Arab/Occidental Asian.

^c^ 1st visit Body Mass Index.

^d^ From October 1st, 2009 to September 30th, 2010 season year = 2010; from October 1st 2010 to September 30th, 2011 season year = 2011; from October 1st 2011 to September 30th 2012, season year = 2012.

^e^ Gonorrhea, *Chlamydia*, Condyloma, herpes.

^f^ Number of days a woman is exposed to the flu season (October–April) between conception date and reference date.

**TABLE 2:** Unadjusted and adjusted association between spontaneous abortion and influenza vaccine during pregnancy.

Outcome	Cases N = 38 (%)	Controls N = 380 (%)	Unadjusted OR (95% CI)	Adjusted^a^ OR (95% CI)
Vaccine exposure	2 (5.3)	29 (7.6)	0.66 (0.15–2.96)	0.53 (0.10–2.68)
Maternal age at reference date^b^, years, mean	33.3 ± 5.5	31.5 ± 4.7	1.08 (1.01–1.16)	**1.10 (1.01–1.19)**
Post-secondary education	32 (84.2)	348 (91.6)	0.48 (0.18–1.26)	0.44 (0.12–1.53)
Household annual income, CAD				
<40,000	4 (10.5)	73 (19.2)	0.64 (0.20–2.02)	0.35 (0.07–1.79)
40,000–80,000	19 (50.0)	127 (33.4)	1.77 (0.87–3.58)	1.86 (0.83–4.20)
≥80,000	15 (39.5)	280 (47.4)	Reference	Reference
Marital status: Living alone	2 (5.3)	24 (6.3)	0.83 (0.19–3.61)	1.17 (0.18–7.55)
Born in Canada	27 (71.1)	278 (73.2)	0.90 (0.44–1.86)	0.81 (0.32–2.05)
Maternal ethnicity - Caucasian	34 (89.5)	321 (84.5)	1.57 (0.54–4.60)	1.75 (0.51–5.99)
Parity				
0	15 (39.5)	205 (54.0)	Reference	Reference
1	17 (44.7)	116 (30.5)	2.02 (0.97–4.22)	1.80 (0.77–4.23)
≥2	6 (15.8)	59 (15.5)	1.37 (0.52–3.63)	1.19 (0.38–3.70)
Body mass index^c^, (kg/m^2^), mean	24.3 ± 5.1	25.1 ± 5.4	0.97 (0.90–1.04)	0.95 (0.89–1.03)
Maternal lifestyle during pregnancy				
Smoking	8 (21.1)	48 (12.6)	1.91 (0.80–4.57)	2.22 (0.78–6.29)
Coffee intake	20 (52.6)	254 (66.8)	0.54 (0.27–1.07)	**0.40 (0.18–0.86)**
Illicit drug	2 (5.3)	13 (3.4)	1.62 (0.33–8.02)	1.19 (0.18–7.93)
Use of assisted reproductive technologies	4 (10.5)	48 (12.6)	0.82 (0.28–2.39)	0.53 (0.15–1.85)
Maternal comorbidities during pregnancy				
Asthma	6 (15.8)	37 (9.7)	1.74 (0.68–4.44)	**2.57 (0.83–7.97)**
Hypertension	2 (5.3)	1 (0.3)	20.0 (1.81–220.6)	-
Hepatitis	1 (2.6)	1 (0.3)	10.0 (0.63–159.9)	14.76 (0.37–590.3)
Gastro-intestinal disease	3 (7.9)	21 (5.3)	1.45 (0.42–5.05)	0.98 (0.18–3.89)
Maternal flu during pregnancy	7 (18.4)	91 (24.0)	0.71 (0.30–1.67)	0.63 (0.24–1.69)
Flu-risk exposure^d^, days, mean ± SD	79.2 ± 49.5	71.5 ± 43.5	1.01 (1.00–1.02)	1.01 (1.00–1.02)

OR, odds ratio; CAD, Canadian dollars; SD, standard deviation.

^a^ Adjusted for maternal age (in continue); post-secondary education; annual household income; marital status; born in Canada; ethnicity; parity; 1^st^ visit body mass index; maternal lifestyle during pregnancy (smoking, coffee intake, illicit drug use); Assisted reproductive technologies use; maternal comorbidities during pregnancy (asthma, hypertension, hepatitis, gastrointestinal disease), maternal flu during pregnancy and before reference date and flu-risk exposure. Diabetes, thyroid disease, anemia, hypercholesterolemia, sexually transmitted diseases and depression were not considered because they have null values for either cases or controls.

^b^ The SA date for the case and end of follow-up (LMP + pregnancy duration) of the control.

^c^ BMI, 1st visit Body Mass Index.

^d^ Number of days a woman is exposed to the flu season (October–April) between conception date and reference date.

Bold numbers indicate p˂0.005.

### Maternal influenza-like symptoms and influenza vaccination exposure

Of the 2,114 individuals included in this sub-cohort, 211 (10.0%) were exposed to the influenza vaccination within 6 months prior to LMP. The prevalence of maternal influenza-like symptoms during the first trimester of pregnancy was 26.5% in the vaccinated group and 25.8% in the unvaccinated group (Supplementary Table S1). Moreover, individuals exposed to the influenza vaccination were more likely to have a higher income than those unvaccinated. Vaccinated individuals were also less likely to smoke and to use assisted reproductive technologies (Supplementary Table S1). After adjusting for potential confounders, there was no significant association between influenza vaccination exposure before LMP and maternal influenza-like symptoms during the first trimester of pregnancy (aOR = 0.92, 95% CI from 0.66 to 1.29) (Table 3).

**TABLE 3:** Unadjusted and adjusted association between influenza during 1^st^ trimester of pregnancy and influenza vaccine before pregnancy.

Characteristics	Flu N = 546 (%)	No flu N = 1,568 (%)	Unadjusted OR (95% CI)	Adjusted^a^ OR (95% CI)
Vaccine exposure	56 (26.5)	155 (73.5)	1.04 (0.76–1.44)	0.92 (0.66–1.29)
Maternal age at first visit^b^, years, mean ± SD	30.6 ± 4.5	31.1 ± 4.6	0.98 (0.96–1.00)	**0.97 (0.95–1.00)**
Gestational age at first visit^b^, weeks, mean ± SD	11.4 ± 1.5	11.1 ± 1.9	**1.10 (1.04–1.17)**	1.04 (0.97–1.11)
Post-secondary education	495 (90.7)	1,397 (89.1)	1.17 (0.84–1.62)	**1.54 (1.05–2.26)**
Annual household income, CAD				
<40,000	83 (15.2)	278 (17.7)	0.82 (0.62–1.09)	0.83 (0.59–1.16)
40,000–80,000	183 (33.5)	485 (30.9)	1.04 (0.83–1.29)	1.01 (0.80–1.28)
≥80,000	280 (51.3)	805 (51.3)	Reference	Reference
Marital status: Living alone	35 (6.4)	79 (4.0)	1.27 (0.85–1.92)	1.54 (0.97–2.44)
Born in Canada	400 (73.3)	1,021 (65.1)	**1.46 (1.18–1.81)**	1.24 (0.95–1.61)
Maternal ethnicity - Caucasian^c^	474 (86.8)	1,302 (83.0)	**1.35 (1.02–1.78)**	1.11 (0.80–1.53)
Parity				
0	265 (48.5)	876 (55.9)	Reference	Reference
1	209 (38.3)	488 (31.1)	**1.42 (1.15–1.75)**	**1.55 (1.24–1.95)**
≥2	72 (13.2)	204 (13.0)	1.17 (0.86–1.58)	1.35 (0.97–1.88)
Body Mass Index^d^, (kg/m^2^), mean ± SD	25.2 ± 5.3	24.9 ± 5.3	1.01 (0.99–1.03)	1.01 (1.00–1.03)
Maternal lifestyle during pregnancy				
Smoking	91 (16.7)	200 (12.8)	1.37 (1.05–1.79)	1.29 (0.96–1.73)
Coffee intake	374 (68.5)	1,031 (65.8)	1.12 (0.91–1.38)	1.07 (0.86–1.34)
Illicit drug	18 (3.3)	52 (3.3)	0.99 (0.58–1.71)	0.87 (0.49–1.53)
Use of assisted reproductive technologies	45 (8.2)	199 (12.7)	0.60 (0.43–0.85)	0.74 (0.52–1.07)
Calendar year of conception				
2010	139 (25.5)	384 (24.5)	Reference	Reference
2011	309 (56.6)	861 (54.9)	0.98 (0.78–1.24)	1.33 (0.88–2.02)
2012	98 (17.9)	323 (20.6)	0.85 (0.63–1.14)	1.07 (0.62–1.86)
Season year of conception^e^				
2010	51 (24.2)	284 (14.9)	Reference	Reference
2011	62 (29.4)	902 (47.4)	0.81 (0.61–1.08)	**0.53 (0.33–0.87)**
2012	98 (45.4)	717 (37.7)	1.06 (0.80–1.41)	0.60 (0.34–1.17)
Maternal comorbidities				
Diabetes	6 (1.1)	9 (0.6)	1.93 (0.68–5.44)	2.25 (0.76–6.68)
Asthma	51 (9.3)	111 (7.1)	1.35 (0.96–1.91)	1.37 (0.95–1.98)
Thyroid disease	26 (4.8)	103 (6.6)	0.70 (0.45–1.09)	0.72 (0.46–1.14)
Anemia	16 (2.9)	50 (3.2)	0.90 (0.50–1.61)	0.98 (0.53–1.80)
Hypercholesterolemia	5 (0.9)	19 (1.2)	0.80 (0.29–2.15)	0.72 (0.26–2.02)
Hypertension	5 (0.9)	17 (1.1)	0.84 (0.31–2.30)	0.67 (0.24–1.89)
Hepatitis	4 (0.7)	12 (0.8)	0.96 (0.31–2.98)	1.07 (0.33–3.43)
Gastro-intestinal disease	42 (7.7)	90 (5.7)	1.37 (0.94–2.00)	1.39 (0.94–2.07)
Sexually transmitted disease^f^	29 (5.3)	67 (4.3)	1.30 (0.84–2.03)	1.20 (0.76–1.89)
Depression	4 (0.7)	13 (0.8)	0.88 (0.29–2.72)	0.93 (0.29–2.95)
Flu-risk exposure^g^, days, mean, ±SD	60.2 ± 30.9	51.4 ± 33.4	**1.01 (1.01–1.01)**	**1.01 (1.01–1.02)**

OR, Odds ratio; CAD, Canadian dollars; SD, standard deviation.

^a^ Adjusted for maternal age (in continue); gestational age (continue) post-secondary education; annual household income; marital status; born in Canada; ethnicity; parity; Body mass index; maternal lifestyle during pregnancy (smoking, coffee intake, illicit drug use); Assisted reproductive technologies use; calendar year of conception; season year of conception, maternal comorbidities during pregnancy (diabetes, asthma, thyroid disease, anemia, hypercholesterolemia, hypertension, hepatitis, gastrointestinal disease; sexually transmitted disease, depression) and flu-risk exposure.

^b^ Date of first questionnaire (first prenatal visit).

^c^ Caucasian = White, East-Asian, South-Asian, Arab/Occidental Asian.

^d^ 1^st^ visit Body Mass Index.

^e^ From October 1st, 2009 to September 30th, 2010 season year = 2010; from October 1st 2010 to September 30th, 2011 season year = 2011; from October 1st 2011 to September 30th 2012, season year = 2012; from October 1st 2012 to September 30th 2013, season year = 2013.

^f^ Gonorrhea, *Chlamydia*, Condyloma, herpes.

^g^ Number of days a woman is exposed to the flu season (October–April) between conception date and visit date.

Bold numbers indicate p˂0.005.

### Birth outcomes and *in utero* influenza vaccine exposure

In this birth cohort, 285 newborns (13.9%) were exposed to the influenza vaccination during pregnancy, while 1,761 (86.1%) were not. In the vaccinated exposed group, the prevalences of the birth outcomes were 7.7%, 9.1%, 4.9% and 12.0% for preterm birth, SGA, LBW and hospitalization during the 1st 3 months of life, respectively (Supplementary Table S2). We also observed that these prevalences were higher in the exposed group than in the non-exposed group, though they were not statistically significant (Supplementary Table S2). After adjustment, none of the outcomes were associated with the influenza vaccine exposure during pregnancy, aOR for preterm birth = 1.30 (95% CI: 0.77–2.19) (Table 4), aOR for SGA = 1.13 (95% CI: 0.71–1.79) (Supplementary Table S3), aOR for LBW = 0.84 (95% CI: 0.31–2.26) (Supplementary Table S4), aOR for all-cause hospitalization = 1.02 (95% CI: 0.65–1.61) (Supplementary Table S5).

**TABLE 4:** Unadjusted and adjusted association between *in-utero* exposure to flu vaccine and preterm birth.

Characteristics	Preterm N = 118 (%)	Full term N = 1928 (%)	Unadjusted OR (95% CI)	Adjusted^a^ OR (95% CI)
Vaccine exposure	22 (18.6)	263 (13.6)	1.45 (0.90–2.35)	1.30 (0.77–2.19)
Baby’s sex – male	62 (52.5)	956 (49.6)	1.12 (0.77–1.63)	1.11 (0.76–1.62)
Maternal age at delivery, years, mean ± SD	31.8 ± 4.7	31.4 ± 4.5	1.02 (0.96–1.06)	1.02 (0.97–1.06)
Post-secondary education	104 (88.1)	1729 (89.7)	0.86 (0.48–1.54)	0.91 (0.47–1.76)
Annual household income, CAD				
<40,000	24 (20.3)	325 (16.9)	1.25 (0.76–2.05)	0.94 (0.52–1.68)
40,000–80,000	37 (31.4)	594 (30.8)	1.12 (0.75–1.71)	0.98 (0.62–1.54)
≥80,000	57 (48.3)	1,009 (52.3)	Reference	Reference
Marital status: Living alone	11 (9.3)	97 (5.0)	1.94 (1.01–3.73)	1.79 (0.86–3.72)
Born in Canada	73 (61.9)	1,299 (67.4)	0.79 (0.54–1.15)	0.73 (0.45–1.18)
Maternal ethnicity - Caucasian^b^	94 (79.7)	1,617 (83.9)	0.76 (0.48–1.20)	0.87 (0.50–1.51)
Parity				
0	69 (58.5)	1,041 (54.0)	Reference	Reference
1	34 (28.8)	637 (33.0)	0.81 (0.53–1.23)	0.76 (0.48–1.79)
≥2	15 (12.7)	250 (13.0)	0.90 (0.51–1.60)	0.74 (0.40–1.40)
Body mass index^c^, (kg/m^2^), mean ± SD	26 0.1 ± 7.4	24.9 ± 5.1	**1.04 (1.01–1.07)**	1.03 (1.00–1.07)
Maternal lifestyle during pregnancy				
Smoking	20 (17.0)	259 (13.4)	1.32 (0.80–2.17)	1.40 (0.82–2.40)
Coffee intake	71 (60.2)	1,295 (67.2)	0.76 (0.52–1.11)	0.76 (0.51–1.14)
Illicit drug	4 (3.4)	64 (3.3)	1.02 (0.37–2.86)	0.98 (0.34–2.83)
Use of assisted reproductive technologies	16 (13.6)	221 (11.5)	1.21 (0.70–2.09)	1.07 (0.59–1.92)
Depression	1 (0.9)	14 (0.7)	1.17 (0.15–8.96)	1.07 (0.13–8.94)
Flu	35 (29.7)	542 (28.1)	1.08 (0.72–1.62)	1.07 (0.70–1.63)
Maternal comorbidities during pregnancy				
Diabetes	3 (2.5)	9 (0.5)	**5.56 (1.49–20.83)**	4.05 (0.88–18.62)
Asthma	9 (7.6)	144 (7.5)	1.02 (0.51–2.06)	1.02 (0.49–2.12)
Thyroid disease	10 (8.5)	117 (6.1)	1.43 (0.73–2.81)	1.39 (0.68–2.83)
Anemia	3 (2.5)	59 (3.1)	0.84 (0.26–2.73)	0.88 (0.30–2.56)
Hypercholesterolemia	1 (0.9)	21 (1.1)	0.78 (0.10–5.82)	0.55 (0.07–4.61)
Hypertension	4 (3.4)	15 (0.8)	4.48 (1.46–13.70)	3.58 (1.05–12.28)
Hepatitis	2 (1.7)	13 (0.7)	2.54 (0.57–11.40)	2.37 (0.51–11.12)
Gastro-intestinal disease	14 (11.9)	114 (5.9)	**2.11 (1.17–3.79)**	**1.93 (1.04–3.56)**
Sexually transmitted disease^d^	7 (5.9)	85 (4.4)	1.37 (0.62–3.03)	1.43 (0.63–3.25)
Depression	1 (0.9)	14 (0.7)	1.17 (0.15–8.96)	1.07 (0.13–8.94)
Flu	35 (29.7)	542 (28.1)	1.08 (0.72–1.62)	1.07 (0.70–1.63)
Season year of delivery^e^				
2011	25 (21.2)	478 (24.8)	Reference	Reference
2012	78 (66.1)	1,049 (54.4)	1.42 (0.90–2.26)	1.34 (0.83–2.16)
2013	15 (12.7)	401 (20.8)	0.72 (0.37–1.38)	0.71 (0.36–1.39)

CAD, Canadian dollars; OR, odd ratio.

^a^ Adjusted for maternal age, education, annual income, marital status, born in Canada, ethnicity, parity, body mass index, maternal lifestyle during pregnancy (smoking, coffee, illicit drug), use of assisted reproductive technologies, maternal comorbidities during pregnancy (diabetes, asthma, thyroid disease, anemia, hypercholesterolemia, hypertension, hepatitis, gastro-intestinal disease, sexually transmitted disease, depression and maternal flu) and season year of delivery.

^b^ Caucasian = White, East-Asian, South-Asian, Arab/Occidental Asian.

^c^ 1st visit Body Mass Index.

^d^ Gonorrhea, *Chlamydia*, Condyloma, herpes.

^e^ From October 1st, 2010 to September 30th, 2011 season year = 2011; from October 1st 2011 to September 30th, 2012 season year = 2012; from October 1st 2012 to September 30th 2013, season year = 2013.

Bold numbers indicate p˂0.005.

### Sensitivity analysis

When restricting the analysis for cases of SA occurring only during influenza season and after adjustment, we found a higher OR of 2.57, though this was not significant (95% CI: 0.36–18.32), when comparing the exposed group to the unexposed group (Supplementary Table S6).

Furthermore, for the neonatal outcomes, we stratified the exposure based on the timing of vaccine administration (6 months prior to LMP, during 1^st^ trimester, during 2^nd^ trimester and during 3^rd^ trimester) and none of the exposures were associated with preterm birth, SGA, LBW or hospitalization during the first 3 months of life (Supplementary Tables S7–S10).

## Discussion

In our study, after adjusting for potential confounders, we found no significant association between the risk of SA following influenza vaccination during pregnancy. Furthermore, no significant association was observed between prenatal influenza vaccination and the occurrence of maternal influenza-like symptoms during pregnancy. In addition, we showed that *in utero* exposure to influenza vaccine was not associated with any of the neonatal outcomes, such as preterm birth, LBW, SGA and hospitalization in the first 3 months of life. Moreover, after adjustments, we found that maternal gastro-intestinal was associated with an increased risk of PTB.

Several studies found no significant association between prenatal influenza vaccine and SA (Donahue et al., 2019; Zhang et al., 2018). Indeed, Zhang et al. (Zhang et al., 2018), in their meta-analysis, found no significant differences between vaccine exposure and the risk of SA. However, Moro et al., using a reporting system of influenza A (H1N1) showed that SA was the most common pregnancy-specific adverse effect; nonetheless this result did not take into account risk factors of SAB especially age, with pregnant persons of 35 years old and more represented around 16% of their sample (Moro et al., 2011). In addition, Donahue et al. (Donahue et al., 2017), in their case-control study in 2017, using two influenza seasons (2010-2011 and 2011-2012), found an increased risk of SA associated with influenza vaccine during pregnancy in the preceding 28 days (aOR of 2.0, 95% CI from 1.1 to 3.6). Nonetheless, using different influenza seasons (2012-13, 2013-14, 2014-15) and the same methodology, Donahue et al in 2019 (Donahue et al., 2019), found no association between prenatal influenza vaccine and spontaneous abortion (aOR of 0.9, 95% CI from 0.6 to 1.5). In addition, Bratton et al., in their metanalysis showed no significant association between SA and prenatal influenza vaccine (Bratton et al., 2014). Our results for SA were then similar to these reviews (Zhang et al., 2018; Bratton et al., 2014) and the case-control study of Donahue et al. (Donahue et al., 2019).

As for prenatal influenza vaccination and the risk of maternal influenza, studies have showed the effectiveness of influenza vaccine for the pregnant individuals and the infants less than 6 months (Getahun et al., 2019; Nunes and Madhi, 2018; Thompson et al., 2019); in our study, there were no association between prenatal influenza vaccine and either the risk of influenza during pregnancy or the risk of hospitalization in children at 3 months. Moreover, some studies reported a significant risk reduction for birth outcomes such as preterm birth (from 13% to 29%) (Giles et al., 2019; Rolfes et al., 2019), LBW (from 18% to 26%) (Giles et al., 2019), associated with the *in utero* exposure of prenatal flu vaccine. Others, reported no significant association between prenatal influenza vaccination and PTB (Jeong et al., 2019; Omer et al., 2020), LBW (Jeong et al., 2019; Omer et al., 2020) and SGA (Giles et al., 2019; Rolfes et al., 2019; Jeong et al., 2019; Omer et al., 2020). In fact, Fell et al., showed that the evidence that maternal influenza vaccine reduces the risk of adverse birth outcomes was conflicting, and this might be due to substantial bias in many observational studies (Fell et al., 2017b). Nonetheless, they showed that the vaccine in pregnant persons was effective to prevent influenza illness (Fell et al., 2017b), as well as Nitsch-Osuch et al. (Nitsch-Osuch et al., 2013). Our study suggests that the influenza vaccine was not significantly associated with any of the newborn outcomes: PTB, LBW, SGA, which is consistent with most of those studies.

While several committees such as the American College of Obstetricians and Gynecologists (Author anonymous, 2018), the NACI recommend influenza vaccine during pregnancy to ensure the pregnant person’s and infant’s protection during influenza season, other studies suggest that data are inconsistent regarding severe illness prevention and birth outcomes. Thus, our results are consistent with the previous recommendations to vaccinate pregnant with the influenza vaccine during any trimester of their pregnancy.

Several studies have shown an increased risk of PTB associated with gastro-intestinal disease. In fact, in their meta-analysis, O’Toole et al., found that inflammatory bowel disease was associated with more than 80% increased odds of PTB (O’Toole et al., 2015). Elbaz et al., also found an increased risk of PTB associated with inflammatory bowel disease (Elbaz et al., 2005). These studies are consistent with our results.

### Strengths and limitations

The strengths of our study include the use of a cohort with follow-ups throughout pregnancy (first, second and third trimesters) and postpartum follow-ups. Additionally, with the inclusion of vaccine dates reported in the questionnaires, we were able to conduct sensitivity analyses stratifying on the timing on the exposure during pregnancy as well as the period of influenza (October–April vs. Mai–September), enhancing the robustness of our findings. On the other hand, we had several limitations. Firstly, we relied on maternal self-reported data to identify prenatal influenza vaccine exposure, which may have led to non-differential misclassification of exposure. However, the same question about the vaccine exposure was asked during follow-ups visits in each trimester, which could have helped to limit the bias, and improve the accuracy of reporting. Moreover, spontaneous abortion was also reported in the questionnaires, and we lacked clinical confirmation to distinguish it from planned or induced abortion, which may introduce social desirability bias. However, this bias is non-differential and would be expected to bias estimates toward the null. Furthermore, for the outcome prenatal maternal influenza, we used maternal self-reported colds and flu-like illness symptoms, which is not a confirmed diagnosis of influenza. This might have overestimated our outcome prevalence; ideally, laboratory-confirmed cases of influenza would have provided a more accurate measure. In fact, in this recent meta-analysis, the authors showed that signs and symptoms have limited accuracy to identify individuals with influenza (Ebell et al., 2025). In addition, our study may be underpowered due to the limited sample size, which could contribute to the wide confidence intervals. For example, we calculated the power *a posteriori* for one of our outcomes. In our cohort assessing vaccination and preterm birth, for our sample of 285 exposed to vaccination during pregnancy (7.7% preterm birth), and 1,761 unexposed (where 5.5% preterm birth) we only had a power of 33%. This could explain our non-significant results in this analysis. Moreover, we did not have information on maternal infection, which might have led to residual confounding.

## Conclusion

Our study has shown that prenatal influenza immunization seemed not to be associated with either the maternal adverse events (SA) nor the birth outcomes (preterm birth, LBW, SGA and all-cause hospitalizations). Moreover, prenatal influenza vaccine seemed not to be associated with the risk of prenatal maternal influenza-like symptoms. Thus, our findings support the safety of maternal influenza vaccination and reinforce current public health recommendations, such as the NACI. Future studies with larger sample sizes and laboratory-confirmed outcomes are needed to confirm these results.

## Data Availability

All data relevant to this study are included in this article or uploaded as Supplementary Material. In order to obtain underlying data from our questionnaires, data are available on request due to privacy/ethical restrictions. Requests to access these datasets should be directed to anick.berard@umontreal.ca.
