# Supplementary material for: Maternal influenza vaccination during pregnancy and the risk of adverse pregnancy and birth outcomes
Source: Front Pharmacol. 2026 Jan 5;16:1691111. doi: 10.3389/fphar.2025.1691111 (PMC12812981; doi:10.3389/fphar.2025.1691111)
Supplement: Supplementary file 1 [file Supplementaryfile1.docx]

Supplementary Material

**Table S1**. Characteristics of study participants according to flu vaccine exposure before pregnancy^*^

| Characteristics | Vaccine   n = 211 (%) | No vaccine  n = 1903 (%) | *p* |
| --- | --- | --- | --- |
| **Influenza during 1^st^ trimester**    Yes    No | 56 (26.5)  155 (73.5) | 490 (25.8)  1413 (74.2) | 0.8032 |
| **Maternal age at first visit^†^,** years, means ±SD     Missing | 31.1±4.1 | 31.0±4.6  5 | 0.6435 |
| **Gestational age at first visit^†^**, weeks, means ±SD | 11.4±1.8 | 11.2±1.8 | 0.1640 |
| **Post-secondary education**      Missing | 197 (93.84)  1 | 1682 (89.0)  14 | 0.0881 |
| **Annual household income**      < 40 000      40 000 – 80 000      ≥ 80 000     Missing | 20 (9.58)  55 (26.18)  130 (61.663.4)  6 | 325 (17.91)  577 (31.8)  911 (50.3)  90 | **0.0006** |
| **Marital status :** Living alone     Missing | 7 (3.3) | 107 (5.6)  3 | 0.1583 |
| **Born in Canada**     Missing | 152 (72.0) | 1265 (66.6)  4 | 0.1115 |
| **Maternal ethnicity -**  Caucasian^‡^     Missing | 180 (85.3) | 1589 (83.8)  7 | 0.5733 |
| **Parity**      0      1      ≥2      Missing | 105 (49.8)  78 (37.0)  28 (13.2) | 1035 (54.4)  619 (32.5)  248 (13.1)  1 | 0.3875 |
| **Body mass index^§^, (kg/m^2^)**, mean ±SD     Missing | 24.9±5.3 | 25.0±5.3  65 | 0.8697 |
| **Maternal lifestyle during pregnancy** |  |  |  |
| Smoking | 18 (8.5) | 273 (14.3) | **0.0200** |
| Coffee intake     Missing | 138 (65.4) | 1262 (66.6)  7 | 0.7353 |
| Illicit drug     Missing | 3 (1.4) | 67 (3.5)  1 | 0.1515 |
| **Use of Assisted Reproductive Technologies**     Missing | 15 (7.1) | 228 (12.0)  1 | **0.0351** |
| **Calendar year of conception**      2010      2011      2012 | 64 (30.3)  89 (42.2)  58 (27.5) | 461 (24.2)  1083 (56.9)  359 (18.9) | **0.0002** |
| **Season year of conception**^\|\|^      2010      2011      2012 | 51 (24.2)  62 (29.4)  98 (45.4) | 284 (14.9)  902 (47.4)  717 (37.7) | **<0.0001** |
|  |  |  | ***Continue*** |

**Table S1**. **Continued**

| Characteristics | Vaccine   n = 211 (%) | No vaccine  n = 1903 (%) | *p* |
| --- | --- | --- | --- |
| **Maternal comorbidities during pregnancy** |  |  |  |
| Diabetes    Missing | 3 (1.4) | 12 (0.6)  5 | 0.1837 |
| Asthma    Missing | 25 (11.9) | 136 (7.2)  10 | 0.0156 |
| Thyroid disease     Missing | 9 (4.3)  1 | 120 (6.4)  14 | 0.2368 |
| Anemia     Missing | 8 (3.8)  4 | 54 (2.9)  65 | 0.4610 |
| Hypercholesterolemia     Missing | 1 (0.5) | 22 (1.2)  19 | 0.7226 |
| Hypertension     Missing | 3 (1.4)  1 | 19 (1.0)  4 | 0.4757 |
| Hepatitis     Missing | 3 (1.4)  2 | 13 (0.7)  3 | 0.2064 |
| Gastro-intestinal disease     Missing | 14 (6.6) | 118 (6.2)  10 | 0.8195 |
| Sexually transmitted disease^¶^     Missing | 8 (3.8)  1 | 88 (4.6)  5 | 0.5855 |
| Depression     Missing | 4 (1.9) | 13 (0.7)  6 | 0.0821 |
| **Flu-risk exposure^#^**, days, mean ±SD | 54.8±33.0 | 53.5±33.0 | 0.5688 |
| ^*^of the 2365, 251 were excluded: 235 had a missing value on vaccination and 16 had a missing value on flu during 1^st^ trimester.  SD: standard deviation, CAD: Canadian dollars.  ^†^ date of first questionnaire (first prenatal visit)  ^‡^ Caucasian= White, East-Asian, South-Asian, Arab/Occidental Asian.  ^§^ 1^st^ visit Body Mass Index.  ^\|\|^ From October 1^st^, 2009 to September 30^th^, 2010 season year = 2010; from October 1^st^ 2010 to September 30^th^, 2011 season year = 2011; from October 1^st^ 2011 to September 30^th^ 2012, season year = 2012; from October 1^st^ 2012 to September 30^th^ 2013, season year = 2013.  ^¶^Gonorrhea, Chlamydia, Condyloma, herpes  ^#^Number of days a woman is exposed to the flu season (October – April) between conception date and reference date. | | | |

**Table S2.** Newborns characteristics according to in-utero flu vaccine exposure*.

| Characteristics | In-utero exposed  N = 285 (%) | In-utero Unexposed  N = 1761 (%) | *p* |
| --- | --- | --- | --- |
| **Sex**       Male       Female       Missing | 145 (50.9)  140 (49.1) | 881 (50.1)  878 (49.9)  2 | 0.8041 |
| **Preterm birth : < 37 weeks** | 22 (7.7) | 96 (5.5) | 0.1276 |
| **Preterm birth : < 34 weeks** | 7 (2.5) | 17 (1.0) | 0.0663 |
| **Small for gestational age -** below 10^th^ percentile        Missing | 26 (9.1) | 149 (8.5)  6 | 0.7235 |
| **Birth weight, mean ±SD, g** | 3325.0 ±532.6 | 3377.6±603.0 | 0.1302 |
| **Low birthweight^†^** | 14 (4.9) | 66 (3.8) | 0.1753 |
| **Hospitalization during the 1^st^ 3 month of life**   - **All cause**   Missing   - **Flu** | 28 (9.812.0)  52  1 (0.4) | 162 (11.5)  353  28 (1.6) | 0.8212  0.1694 |
| ^*^of the 2365, 263 were excluded: 235 had a missing value on vaccination, 72 had missing values on birth date and 12 were stillbirths; n = 2046.  SD, standard deviation.  ^†^ Weight < 2500g.  In-utero is considered exposed to flu vaccine when the mother received the flu shot the same season of infant birth date. | | | |

**Table S3.** Unadjusted and adjusted association between in-utero exposure to flu vaccine and **Small for gestational age**^*^.

| Characteristics | SGA  N = 175 (%) | No SGA  N = 1865 (%) | Unadjusted  OR  (95 % CI) | Adjusted†   OR (95 % CI) |
| --- | --- | --- | --- | --- |
| **Vaccine exposure** | 26 (14.9) | 259 (13.9) | 1.08 (0.70 - 1.68) | 1.13 (0.71 - 1.79) |
| **Baby’s sex –** male | 84 (48.0) | 932 (50.0) | 0.92 (0.68 - 1.26) | 0.92 (0.67 - 1.26) |
| **Maternal age at delivery,** years, mean ±SD | 31.2±5.0 | 31.5±4.5 | 0.99 (0.95 - 1.02) | 1.02 (0.98 - 1.06) |
| **Gestational age at delivery, weeks, mean** ±SD | 39.2±1.9 | 39.1±1.8 | 1.02 (0.93 - 1.11) | 1.02 (0.93 - 1.12) |
| **Post-secondary education** | 149 (85.1) | 1679 (90.0) | **0.64 (0.41 - 1.00)** | 0.68 (0.40 - 1.14) |
| **Annual household income, CAD**      < 40 000      40 000 – 80 000      ≥ 80 000 | 35 (20.0)  50 (28.6)  90 (51.4) | 313 (16.8)  579 (31.0)  973 (52.2) | 1.21 (0.80 - 1.82)  0.93 (0.65 - 1.34)  reference | 1.14 (0.69 - 1.87)  0.92 (0.62 - 1.35)  Reference |
| **Marital status :** Living alone | 13 (7.4) | 95 (5.1) | 1.50 (0.82 - 2.73) | 1.09 (0.56 - 2.12) |
| **Born in Canada** | 118 (67.4) | 1250 (67.0) | 1.02 (0.73 - 1.42) | 0.90 (0.60 - 1.36) |
| **Maternal ethnicity -**  Caucasian^‡^ | 145 (82.9) | 1563 (83.8) | 0.93 (0.62 - 1.41) | 0.79 (0.49 - 1.28) |
| **Parity**      0      1      ≥2 | 121 (69.1)  40 (22.9)  14 (8.0) | 987 (52.9)  627 (33.6)  251 (13.5) | Reference  **0.52 (0.36 - 0.75)**  **0.46 (0.26 - 0.81)** | Reference  **0.51 (0.34 - 0.75)**  **0.43 (0.24 - 0.79)** |
| **Body mass index**^§^**,** (kg/m^2^), mean ±SD | 24.1±4.6 | 25.0±5.3 | **0.96 (0.93 - 0.99)** | **0.96 (0.93 - 0.99)** |
| **Maternal lifestyle during pregnancy** |  |  |  |  |
| Smoking | 41 (23.4) | 237 (12.7) | **2.10 (1.44 - 3.06)** | **1.99 (1.32 - 2.99)** |
| Coffee intake | 117 (66.9) | 1244 (66.7) | 1.01 (0.73 - 1.40) | 0.91 (0.65 - 1.29) |
| Illicit drug | 9 (5.1) | 59 (3.2) | 1.66 (0.81 - 3.41) | 1.10 (0.51 - 2.34) |
| **Use of Assisted reproductive technologies** | 18 (10.3) | 219 (11.7) | 0.86 (0.52 - 1.43) | 0.75 (0.43 - 1.28) |
| **Maternal comorbidities during pregnancy** |  |  |  |  |
| Diabetes | 1 (0.6) | 11 (0.6) | 0.97 (0.12 - 7.55) | 1.37 (0.16 - 11.73) |
| Asthma | 22 (12.6) | 130 (7.0) | **1.92 (1.19 - 3.11)** | **1.82 (1.10 - 3.00)** |
| Thyroid disease | 12 (6.9) | 114 (6.1) | 1.13 (0.61 - 2.09) | 1.32 (0.70 - 2.51) |
| Anemia | 4 (2.3) | 56 (3.0) | 0.76 (0.27 - 2.11) | 0.89 (0.34 - 2.30) |
| Hypercholesterolemia | 1 (0.6) | 20 (1.1) | 0.53 (0.07 - 3.97) | 0.55 (0.07 - 4.21) |
| Hypertension | 3 (1.7) | 16 (0.9) | 2.02 (0.58 - 6.99) | 2.88 (0.76 - 10.87) |
| Hepatitis | 1 (0.6) | 14 (0.8) | 0.76 (0.10 - 5.81) | 0.86 (0.11 - 6.93) |
| Gastro-intestinal disease^\|\|^ | 11 (6.3) | 116 (6.2) | 1.01 (0.53 - 1.92) | 1.03 (0.54 - 1.98) |
| Sexually transmitted disease | 12 (6.9) | 80 (4.3) | 1.64 (0.88 - 3.08) | 1.32 (0.69 - 2.54) |
| Flu | 51 (29.1) | 525 (28.2) | 1.05 (0.75 - 1.48) | 1.09 (0.77 - 1.55) |
| **Season year of delivery^¶^**       2011       2012       2013 | 50 (28.5)  89 (50.9)  36 (20.6) | 452 (24.2)  1032 (55.4)  379 (20.4) | Reference  0.78 (0.54 - 1.12)  0.86 (0.55 - 1.35) | Reference  0.79 (0.54 - 1.15)  0.86 (0.54 - 1.38) |
| SD: standard deviation, CAD: Canadian dollars; OR, odd ratio  *6 values deleted due to missing on the outcome; **N = 2040.**  **†**Adjusted for maternal age, gestational age, education, annual income, marital status, born in Canada, ethnicity, parity, body mass index, maternal lifestyle during pregnancy (smoking, coffee, illicit drug), use of assisted reproductive technologies, maternal comorbidities during pregnancy (diabetes, asthma, thyroid disease, anemia, hypercholesterolemia, hypertension, hepatitis, gastro-intestinal disease, sexually transmitted disease and maternal flu) and season year of delivery.  ^‡^ Caucasian= White, East-Asian, South-Asian, Arab/Occidental Asian.  ^§^ 1^st^ visit Body Mass Index.  ^\|\|^ Gonorrhea, Chlamydia, Condyloma, herpes  ^¶ -^From October 1^st^, 2010 to September 30^th^, 2011 season year = 2011; from October 1^st^ 2011 to September 30^th^, 2012 season year = 2012; from October 1^st^ 2012 to September 30^th^ 2013, season year = 2013. | | | | |

**Table S4**. Unadjusted and adjusted association between in-utero exposure to flu vaccine and low birth weight

| Characteristics | Low birth weight  N = 80 (%) | Normal birth weight  N = 1966 (%) | Unadjusted  OR  (95 % CI) | Adjusted*   OR (95 % CI) |
| --- | --- | --- | --- | --- |
| **Vaccine exposure** | 14 (17.5) | 271 (13.8) | 1.33 (0.74 - 2.40) | 0.84 (0.31 - 2.26) |
| **Baby’s sex –** male | 50 (62.5) | 698 (49.2) | **1.72 (1.08 - 2.73)** | **2.93 (1.47 - 5.83)** |
| **Maternal age at delivery,** years, mean ±SD | 31.8±4.7 | 31.5±4.5 | 1.02 (0.97 - 1.07) | 1.03 (0.95 - 1.11) |
| **Gestational age at delivery, weeks,** mean ±SD | 34.5±3.8 | 39.3±1.4 | **0.31 (0.26 - 0.38)** | **0.28 (0.23 - 0.35)** |
| **Post-secondary education** | 67 (83.8) | 1766 (89.8) | 0.58 (0.32 - 1.08) | 0.57 (0.21 - 1.53) |
| **Annual household income, CAD**      < 40 000      40 000 – 80 000      ≥ 80 000 | 12 (15.0)  30 (37.5)  38 (47.5) | 337 (17.1)  601 (30.6)  1028 (52.3) | 0.96 (0.50 - 1.87)  1.35 (0.83 - 2.20)  reference | 1.11 (0.40 - 3.08)  1.05 (0.49 - 2.23)  Reference |
| **Marital status :** Living alone | 7 (8.8) | 101 (5.1) | 1.77 (0.80 - 3.94) | 1.17 (0.31 - 4.39) |
| **Born in Canada** | 52 (65.0) | 1320 (67.1) | 0.91 (0.57 - 1.45) | 1.44 (0.62 - 3.34) |
| **Maternal ethnicity -**  Caucasian^†^ | 65 (81.3) | 1646 (83.7) | 0.84 (0.47 - 1.50) | 1.00 (0.37 - 2.69) |
| **Parity**      0      1      ≥2 | 48 (60.0)  23 (28.8)  9 (11.2) | 1062 (54.0)  648 (33.0)  256 (13.0) | Reference  0.79 (0.47 - 1.30)  0.78 (0.38 - 1.61) | Reference  0.52 (0.24 - 1.13)  0.62 (0.21 - 1.81) |
| **Body mass index^‡^,** (kg/m^2^), mean ±SD | 24.7 (5.6) | 24.9 (5.3) | 0.99 (0.85 - 1.03) | **0.93 (0.88 - 0.99)** |
| **Maternal lifestyle during pregnancy** |  |  |  |  |
| Smoking | 16 (20.0) | 263 (13.4) | 1.62 (0.92 - 2.84) | 1.41 (0.60 - 3.34) |
| Coffee intake | 50 (63.3) | 1316 (66.9) | 0.82 (0.52 - 1.31) | 0.84 (0.42 - 1.66) |
| Illicit drug | 2 (2.5) | 66 (3.4) | 0.74 (0.18 - 3.07) | 0.39 (0.04 - 4.35) |
| **Use of Assisted reproductive technologies** | 13 (16.3) | 224 (11.4) | 1.51 (0.82 - 2.78) | 0.83 (0.31 - 2.22) |
| **Maternal comorbidities during pregnancy** | | | | |
| Diabetes | 2 (2.5) | 10 (0.5) | 5.02 (1.08 - 23.28) | 1.94 (0.11 - 35.73) |
| Asthma | 8 (10.0) | 145 (7.4) | 1.40 (0.66 - 2.95) | 1.75 (0.59 - 5.20) |
| Thyroid disease | 8 (10.0) | 119 (6.1) | 1.73 (0.81 - 3.66) | 1.99 (0.60 - 6.57) |
| Anemia | 2 (2.5) | 60 (3.1) | 0.82 (0.20 - 3.39) | 0.23 (0.02 -3.07) |
| Hypercholesterolemia | 0 (0.0) | 22 (1.1) | - | - |
| Hypertension | 3 (3.8) | 16 (0.8) | 4.75 (1.36 - 16.64) | 1.29 (0.17 - 10.01) |
| Hepatitis | 2 (2.5) | 13 (0.7) | 3.85 (0.86 - 17.37) | 4.50 (0.29 - 69.99) |
| Gastro-intestinal disease | 3 (3.8) | 125 (6.4) | 0.57 (0.18 - 1.84) | 0.22 (0.03 - 1.48) |
| Sexually transmitted disease^§^ | 2 (2.5) | 90 (4.6) | 0.53 (0.13 - 2.21) | 0.50 (0.09 - 2.70) |
| Depression | 1 (1.3) | 14 (0.7) | 1.77 (0.23 - 13.59) | 2.11 (0.11 - 41.63) |
| Flu | 23 (28.8) | 554 (28.2) | 1.03 (0.63 - 1.69) | 0.88 (0.42 - 1.86) |
| **Season year of delivery**^\|\|^       2011       2012       2013 | 21 (26.3)  48 (60.0)  11 (13.7) | 482 (24.5)  1079 (54.9)  405 (20.6) | Reference  1.02 (0.61 - 1.72)  0.62 (0.30 - 1.31) | Reference  1.08 (0.48 - 2.42)  0.78 (0.26 - 2.32) |
| **SD, standard deviation; CAD, Canadian dollars. OR, odd ratio**  *****Adjusted for maternal age, gestational age, education, annual income, marital status, born in Canada, ethnicity, parity, body mass index, maternal lifestyle during pregnancy (smoking, coffee, illicit drug), use of assisted reproductive technologies, maternal comorbidities during pregnancy (diabetes, asthma, thyroid disease, anemia, hypertension, hepatitis, gastro-intestinal disease, sexually transmitted disease, depression and maternal flu) and season year of delivery.  ^-^From October 1^st^, 2010 to September 30^th^, 2011 season year = 2011; from October 1^st^ 2011 to September 30^th^, 2012 season year = 2012; from October 1^st^ 2012 to September 30^th^ 2013, season year = 2013.  ^†^ Caucasian= White, East-Asian, South-Asian, Arab/Occidental Asian.  ^‡^ 1^st^ visit Body Mass Index.  ^§^Gonorrhea, Chlamydia, Condyloma, herpes  ^\|\|-^From October 1^st^, 2010 to September 30^th^, 2011 season year = 2011; from October 1^st^ 2011 to September 30^th^, 2012 season year = 2012; from October 1^st^ 2012 to September 30^th^ 2013, season year = 2013. | | | | |

**Table S5**. Unadjusted and adjusted association between in-utero exposure to flu vaccine and all cause hospitalisation*.

| Characteristics | Hospitalization  N = 190 (%) | No hospitalization  N = 1451 (%) | Unadjusted  OR  (95 % CI) | Adjusted^†^   OR (95 % CI) |
| --- | --- | --- | --- | --- |
| **Vaccine exposure** | 28 (14.7) | 205 (14.1) | 1.05 (0.69 - 1.61) | 1.02 (0.65 - 1.61) |
| **Baby’s sex –** male | 81 (42.6) | 735 (50.7) | **0.72 (0.53 - 0.98)** | 0.73 (0.54 - 1.00) |
| **Maternal age at delivery,** years, mean ±SD | 31.8±4.3 | 31.4±4.4 | 1.02 (0.99 - 1.06) | 1.01 (0.97 - 1.05) |
| **Gestational age at delivery, weeks,** mean ±SD | 38.6±2.2 | 39.2±1.6 | **0.83 (0.77 - 0.90)** | **0.82 (0.75 - 0.90)** |
| **Post-secondary education** | 178 (93.7) | 1318 (90.8) | 1.50 (0.81 - 2.76) | 1.56 (0.80 - 3.05) |
| **Annual household income, CAD**      < 40 000      40 000 – 80 000      ≥ 80 000 | 28 (14.7)  63 (33.2)  99 (52.1) | 218 (15.0)  429 (29.6)  804 (55.4) | 1.04 (0.67 - 1.63)  1.19 (0.85 - 1.67)  reference | 1.32 (0.78 - 2.22)  1.27 (0.88 - 1.83)  Reference |
| **Marital status :** Living alone | 6 (3.2) | 73 (5.0) | 0.62 (0.26 - 1.44) | 0.57 (0.23 - 1.43) |
| **Born in Canada** | 131 (69.0) | 1023 (70.5) | 0.93 (0.67 - 1.29) | 1.03 (0.68 - 1.56) |
| **Maternal ethnicity -**  Caucasian^‡^ | 161 (84.7) | 1244 (85.7) | 0.92 (0.61 - 1.41) | 1.05 (0.64 - 1.73) |
| **Parity**      0      1      ≥2 | 86 (45.3)  79 (41.6)  25 (13.1) | 831 (57.3)  454 (31.3)  166 (11.4) | Reference  **1.68 (1.21 - 2.33)**  1.46 (0.81 - 2.34) | Reference  **1.59 (1.12 - 2.26)**  1.32 (0.79 - 2.20) |
| **Body mass index**^§^**,** (kg/m^2^), mean ±SD | 25.2 (4.9) | 24.9 (5.2) | 1.01 (0.99 - 1.04) | 1.00 (0.97 - 1.03) |
| **Maternal lifestyle during pregnancy** |  |  |  |  |
| Smoking | 18 (9.5) | 201 (13.9) | 0.65 (0.39 - 1.08) | 0.70 (0.41 - 1.20) |
| Coffee intake | 127 (66.8) | 983 (67.8) | 0.96 (0.70 - 1.32) | 1.06 (0.75 - 1.48) |
| Illicit drug | 5 (2.6) | 47 (3.2) | 0.81 (0.32 - 2.06) | 0.97 (0.36 - 2.59) |
| **Use of Assisted reproductive technologies** | 16 (8.4) | 173 (11.9) | 0.68 (0.40 - 1.16) | 0.62 (0.35 - 1.11) |
| **Maternal comorbidities during pregnancy** | | | | |
| Diabetes | 3 (1.6) | 6 (0.4) | 3.86 (0.96 - 15.58) | 3.03 (0.67 - 13.70) |
| Asthma | 16 (8.4) | 111 (7.7) | 1.10 (0.64 - 1.92) | 1.29 (0.73 - 2.28) |
| Thyroid disease | 13 (6.8) | 92 (6.3) | 1.09 (0.60 - 1.98) | 1.19 (0.63 - 2.24) |
| Anemia | 2 (1.1) | 37 (2.6) | 0.41 (0.10 - 1.70) | 0.31 (0.07 - 1.32) |
| Hypercholesterolemia | 0 (0.0) | 17 (1.2) | - | - |
| Hypertension | 1 (0.5) | 14 (1.0) | 0.54 (0.07 - 4.15) | 0.31 (0.04 - 2.52) |
| Hepatitis | 4 (2.1) | 7 (0.5) | **4.44 (1.29 - 15.30)** | **4.54 (1.24 - 16.63)** |
| Gastro-intestinal disease | 15 (7.9) | 87 (6.0) | 1.34 (0.76 - 2.38) | 1.44 (0.79 - 2.60) |
| Sexually transmitted disease^\|\|^ | 11 (5.8) | 68 (4.7) | 1.25 (0.65 - 2.41) | 1.33 (0.67 - 2.63) |
| Depression | 0 (0.0) | 10 (0.7) | - | - |
| **Maternal flu during pregnancy** | 58 (30.5) | 418 (28.8) | 1.09 (0.78 - 1.51) | 1.02 (0.73 - 1.44) |
| **Season year of delivery^¶^**       2011       2012       2013 | 39 (20.5)  102 (53.7)  49 (25.8) | 310 (21.4)  851 (58.6)  290 (20.0) | Reference  0.95 (0.64 - 1.41)  1.34 (0.86 - 2.11) | Reference  1.04 (0.69 - 1.55)  **1.54 (1.05 - 2.27)** |
| **SD, standard deviation; CAD, Canadian dollars. OR, odd ratio**  *405 values deleted due to missing on the outcome, **N = 1641.**  **†**Adjusted for maternal age, gestational age, education, annual income, marital status, born in Canada, ethnicity, parity, body mass index, maternal lifestyle during pregnancy (smoking, coffee, illicit drug), use of assisted reproductive technologies, maternal comorbidities during pregnancy (diabetes, asthma, thyroid disease, anemia, hypertension, hepatitis, gastro-intestinal disease, sexually transmitted disease, maternal flu) and season year of delivery.  ^‡^ Caucasian= White, East-Asian, South-Asian, Arab/Occidental Asian.  ^§^ 1^st^ visit Body Mass Index.  ^\|\|^Gonorrhea, Chlamydia, Condyloma, herpes  ^¶^From October 1^st^, 2010 to September 30^th^, 2011 season year = 2011; from October 1^st^ 2011 to September 30^th^, 2012 season year = 2012; from October 1^st^ 2012 to September 30^th^ 2013, season year = 2013. | | | | |

**Controls match on cases LMP (+/- 15 days) and conception season ^*^,**

**N = 300**

**End of pregnancy not during vaccine period (October – April)**

**N = 30**

End of pregnancy not during vaccine period (Mai – September)

N = 8

Potential controls,

N = 2076

Cases of spontaneous abortion, n = 38

2,114

14 excluded:

- 2 (14.3 %) elective abortions,

- 1 (7.1 %) molar pregnancy,

- 11 (78.6 %) therapeutic termination

2,128

237 excluded:

-4 (1.7 %) no data on the pregnancy outcome

-233 (98.3 %) no data on the vaccine exposure

2,365 recruited pregnant women

## Figure **S1**. Flow chart describing the selection of spontaneous abortion cases happened during seasonal vaccine period and controls. *Season year of vaccine period (e.g. from October 1st, 2010 to September 30th, 2011, season year is 2011). LMP: last menstrual period. SA: Spontaneous abortion

**Different definitions of influenza vaccine exposure depending on the outcome**

Exposed if vaccine during this period

- 12 months

Reference date

- 6 months

LMP date

**Figure S1.a** Influenza vaccine exposure definition for spontaneous abortion.

Exposed if vaccine during this period

End of pregnancy date

- 6 months

- 12 months

LMP date

**Figure S1.b** Influenza vaccine exposure definition for maternal influenza during pregnancy.

Exposed if vaccine during this period

- 12 months

- 6 months

End of pregnancy date

LMP date

**Figure S1.c** Influenza vaccine exposure definition for newborns’ outcomes.

**Table S6**. **Unadjusted and adjusted association between spontaneous abortion and influenza vaccine during pregnancy**

| Characteristics | Cases  N = 30 (%) | Controls  N = 300 (%) | Unadjusted OR  (95 % CI) | Adjusted* OR  (95 % CI) |
| --- | --- | --- | --- | --- |
| **Vaccine exposure** | 2 (6.7) | 15 (5.0) | 1.37 (0.30 - 6.54) | 2.57 (0.36 - 18.32) |
| **Maternal age at reference date^†^,** years | 32.9 (5.0) | 31.2 (4.7) | 1.07 (0.94 - 1.16) | **1.19 (1.06 - 1.33)** |
| **Post-secondary education** | 26 (86.7) | 275 (91.7) | 0.60 (0.20 - 1.82) | 0.54 (0.11 - 2.79) |
| **Household annual income,** CAD  < 40 000  40 000 – 80 000  ≥ 80 000 | 3 (10.0)  15 (50.0)  12 (40.0) | 71 (23.7)  95 (31.7)  134 (44.7) | 0.47 (0.13 - 1.72)  1.72 (0.79 - 3.76)  reference | 0.38 (0.06 - 2.44)  2.30 (0.86 - 6.17)  reference |
| **Marital status :** Living alone | 2 (6.7) | 26 (8.7) | 0.75 (0.17 - 3.34) | 0.93 (0.14 - 5.98) |
| **Born in Canada** | 23 (76.7) | 196 (65.3) | 1.75 (0.73 - 4.22) | 1.71 (0.47 - 6.20) |
| **Maternal Ethnicity -** Caucasian^‡^ | 27 (90.0) | 245 (81.7) | 1.99 (0.59 - 6.71) | 2.41 (0.39 - 14.69) |
| **Parity**  0  1  ≥2 | 11 (36.7)  13 (43.3)  6 (20.0) | 156 (52.0)  109 (36.3)  35 (11.7) | reference  1.70 (0.73 - 3.93)  2.41 (0.84 - 6.91) | Reference  1.40 (0.48 - 4.05)  1.88 (0.53 - 6.68) |
| **Body Mass Index^§^,** (kg/m^2^), mean | 24.6 (5.1) | 25.1 (5.4) | 0.98 (0.90 - 1.06) | 0.95 (0.86 - 1.05) |
| **Maternal lifestyle during pregnancy** |  |  |  |  |
| Smoking | 8 (26.7) | 50 (16.7) | 1.83 (0.77 - 4.39) | 2.26 (0.76 - 6.71) |
| Coffee intake | 17 (56.7) | 209 (69.6) | 0.56 (0.26 - 1.23) | 0.34 (0.13 - 0.95) |
| Illicit drug | 2 (6.7) | 8 (2.7) | 2.57 (0.53 - 12.52) | 3.34 (0.41 - 27.20) |
| **Use of Assisted Reproductive Technologies** | 3 (10.0) | 39 (13.0) | 0.75 (0.22 - 2.55) | 0.67 (0.14 - 3.14) |
| **Maternal comorbidities during pregnancy** | | | | |
| Asthma | 4 (13.3) | 26 (8.7) | 1.58 (0.53 - 4.74) | 2.73 (0.68 - 10.93) |
| Hepatitis | 1 (3.3) | 2 (0.7) | 5.00 (0.45 - 55.14) | 22.24 (0.95 - 521.61) |
| Gastro-intestinal disease | 2 (6.7) | 14 (4.7) | 1.44 (0.32 - 6.40) | 1.42 (0.24 - 8.43) |
| **Maternal influenza during pregnancy** | 7 (23.3) | 60 (20.0) | 1.22 (0.50 - 3.00) | 0.92 (0.30 - 2.80) |
| **Flu-risk exposure**^\|\|^, days, mean ±SD | 92.0 (42.1) | 69.02 (44.1) | **1.02 (1.01 - 1.03)** | **1.03 (1.01 - 1.04)** |
| OR: odds ratio; CAD: Canadian dollars.  * Adjusted for maternal age (in continue); post-secondary education; annual household income; marital status; born in Canada; ethnicity; parity; 1^st^ visit body mass index; maternal lifestyle during pregnancy (smoking, coffee intake, illicit drug use); Assisted reproductive technologies use; maternal comorbidities during pregnancy (asthma, hepatitis, gastrointestinal disease, depression), maternal flu during pregnancy and before reference date and flu-risk exposure. Diabetes, thyroid disease, anemia, hypercholesterolemia, hypertension, depression, sexually transmitted diseases were removed from the model because they did not have enough values for either cases or controls.  ^†^the SA date for the case and end of follow-up (LMP + pregnancy duration) of the control.  ‡Caucasian= White, East-Asian, South-Asian, Arab/Occidental Asian.  ^§^ BMI, 1^st^ visit Body Mass Index.  ^\|\|^Number of days a woman is exposed to the flu season (October – April) between conception date and reference date. | | | | |

## Table S7. Unadjusted and adjusted association between in-utero patterns of exposure to flu vaccine and prematurity.

| Characteristics | Preterm  N = 118 (%) | Full term  N = 1928 (%) | Unadjusted  OR  (95 % CI) | Adjusted*   OR (95 % CI) |
| --- | --- | --- | --- | --- |
| **Vaccine exposure patterns**        No exposure        Exposure 6 months before LMP        Exposure during 1^st^ trimester        Exposure during 2^nd^ trimester        Exposure during 3^rd^ trimester | 96 (81.4)  6 (5.1)  10 (8.5)  4 (3.4)  2 (1.6) | 1665 (86.4)  48 (2.5)  95 (4.9)  97 (5.0)  23 (1.2) | Reference  2.17 (0.91 - 5.19)  1.83 (0.92 - 3.62)  0.72 (0.26 - 1.99)  1.51 (0.35 - 6.49) | Reference  1.77 (0.70 - 4.45)  1.57 (0.74 - 3.34)  0.68 (0.24 - 1.94)  1.74 (0.39 - 7.76) |
| **Baby’s sex –** male | 62 (52.5) | 956 (49.6) | 1.12 (0.77 - 1.63) | 1.10 (0.75 - 1.61) |
| **Maternal age at delivery,** years, mean ±SD | 31.8±4.7 | 31.4±4.5 | 1.02 (0.96 - 1.06) | 1.02 (0.97 - 1.06) |
| **Post-secondary education** | 104 (88.1) | 1729 (89.7) | 0.86 (0.48 - 1.54) | 0.93 (0.48 - 1.80) |
| **Annual household income, CAD**      < 40 000      40 000 – 80 000      ≥ 80 000 | 24 (20.3)  37 (31.4)  57 (48.3) | 325 (16.9)  594 (30.8)  1009 (52.3) | 1.25 (0.76 - 2.05)  1.12 (0.75 - 1.71)  reference | 0.96 (0.52 - 1.76)  1.03 (0.65 - 1.62)  Reference |
| **Marital status :** Living alone | 11 (9.3) | 97 (5.0) | 1.94 (1.01 - 3.73) | 1.81 (0.87 - 3.78) |
| **Born in Canada** | 73 (61.9) | 1299 (67.4) | 0.79 (0.54 - 1.15) | 0.73 (0.45 -  1.18) |
| **Maternal ethnicity -**  Caucasian^†^ | 94 (79.7) | 1617 (83.9) | 0.76 (0.48 - 1.20) | 0.88 (0.51 - 1.52) |
| **Parity**      0      1      ≥2 | 69 (58.5)  34 (28.8)  15 (12.7) | 1041 (54.0)  637 (33.0)  250 (13.0) | Reference  0.81 (0.53 - 1.23)  0.90 (0.51 - 1.60) | Reference  0.76 (0.48 - 1.19)  0.74 (0.39 - 1.40) |
| **Body mass index^‡^,** (kg/m^2^), mean ±SD | 26 .1 (7.4) | 24.9 (5.1) | **1.04 (1.01 - 1.07)** | 1.03 (1.00 - 1.07) |
| **Maternal lifestyle during pregnancy** |  |  |  |  |
| Smoking | 20 (17.0) | 259 (13.4) | 1.32 (0.80 - 2.17) | 1.40 (0.81 - 2.40) |
| Coffee intake | 71 (60.2) | 1295 (67.2) | 0.76 (0.52 - 1.11) | 0.77 (0.52 - 1.15) |
| Illicit drug | 4 (3.4) | 64 (3.3) | 1.02 (0.37 - 2.86) | 0.96 (0.33 - 2.78) |
| **Use of Assisted reproductive technologies** | 16 (13.6) | 221 (11.5) | 1.21 (0.70 - 2.09) | 1.06 (0.59 - 1.91) |
| **Maternal comorbidities during pregnancy** |  |  |  |  |
| Diabetes | 3 (2.5) | 9 (0.5) | **5.56 (1.49 - 20.83)** | 3.80 (0.82 - 17.62) |
| Asthma | 9 (7.6) | 144 (7.5) | 1.02 (0.51 - 2.06) | 1.03 (0.49 - 2.14) |
| Thyroid disease | 10 (8.5) | 117 (6.1) | 1.43 (0.73 - 2.81) | 1.43 (0.70 - 2.93) |
| Anemia | 3 (2.5) | 59 (3.1) | 0.84 (0.26 - 2.73) | 0.88 (0.30 - 2.56) |
| Hypercholesterolemia | 1 (0.9) | 21 (1.1) | 0.78 (0.10 - 5.82) | 0.55 (0.07 - 4.67) |
| Hypertension | 4 (3.4) | 15 (0.8) | **4.48 (1.46 - 13.70)** | 3.14 (0.89 - 11.07) |
| Hepatitis | 2 (1.7) | 13 (0.7) | 2.54 (0.57 - 11.40) | 2.29 (0.48 - 11.01) |
| Gastro-intestinal disease | 14 (11.9) | 114 (5.9) | **2.11 (1.17 - 3.79)** | **1.95 (1.06 - 3.62)** |
| Sexually transmitted disease^§^ | 7 (5.9) | 85 (4.4) | 1.37 (0.62 - 3.03) | 1.48 (0.65 - 3.36) |
| Depression | 1 (0.9) | 14 (0.7) | 1.17 (0.15 - 8.96) | 1.04 (0.12 - 8.80) |
| **Maternal flu during pregnancy** | 35 (29.7) | 542 (28.1) | 1.08 (0.72 - 1.62) | 1.06 (0.70 - 1.61) |
| **Season year of delivery**^\|\|^       2011       2012       2013 | 25 (21.2)  78 (66.1)  15 (12.7) | 478 (24.8)  1049 (54.4)  401 (20.8) | Reference  1.42 (0.90 - 2.26)  0.72 (0.37 - 1.38) | Reference  1.34 (0.83 - 2.16)  0.70 (0.35 - 1.38) |
| **CAD, Canadian dollars. OR, odd ratio**  *****Adjusted for maternal age, education, annual income, marital status, born in Canada, ethnicity, parity, body mass index, maternal lifestyle during pregnancy (smoking, coffee, illicit drug), use of assisted reproductive technologies, maternal comorbidities during pregnancy (diabetes, asthma, thyroid disease, anemia, hypercholesterolemia, hypertension, hepatitis, gastro-intestinal disease, sexually transmitted disease, depression and maternal flu) and season year of delivery.  †Caucasian= White, East-Asian, South-Asian, Arab/Occidental Asian.  ^‡^BMI, 1^st^ visit Body Mass Index.  ^§^Gonorrhea, Chlamydia, Condyloma, herpes  ^\|\|-^From October 1^st^, 2010 to September 30^th^, 2011 season year = 2011; from October 1^st^ 2011 to September 30^th^, 2012 season year = 2012; from October 1^st^ 2012 to September 30^th^ 2013, season year = 2013. | | | | |

## Table S8. Unadjusted and adjusted association between in-utero patterns of exposure to flu vaccine and Small for gestational age^*^.

| Characteristics | SGA  N = 175 (%) | No SGA  N = 1865 (%) | Unadjusted  OR  (95 % CI) | Adjusted^†^   OR (95 % CI) |
| --- | --- | --- | --- | --- |
| **Vaccine exposure patterns**        No exposure        Exposure 6 months before LMP        Exposure during 1^st^ trimester        Exposure during 2^nd^ trimester        Exposure during 3^rd^ trimester | 149 (85.1)  7 (4.0)  7 (4.0)  12 (11.9)  0 (0.0) | 1606 (86.1)  47 (2.5)  98 (5.3)  89 (4.8)  25 (1.3) | Reference  1.61 (0.71 - 3.61)  0.77 (0.35 - 1.67)  1.45 (0.78 - 2.72)  - | Reference  1.76 (0.75 - 4.13)  0.86 (0.38 - 1.95)  1.55 (0.80 - 2.99)  - |
| **Baby’s sex –** male | 84 (48.0) | 932 (50.0) | 0.92 (0.68 - 1.26) | 0.93 (0.67 - 1.28) |
| **Maternal age at delivery,** years, mean ±SD | 31.2±5.0 | 31.5±4.5 | 0.99 (0.95 - 1.02) | 1.02 (0.98 - 1.06) |
| **Gestational age at delivery, weeks,** mean ±SD | 39.2±1.9 | 39.1±1.8 | 1.02 (0.93 - 1.11) | 1.02 (0.94 - 1.12) |
| **Post-secondary education** | 149 (85.1) | 1679 (90.0) | **0.64 (0.41 - 1.00)** | 0.68 (0.40 - 1.14) |
| **Annual household income, CAD**      < 40 000      40 000 – 80 000      ≥ 80 000 | 35 (20.0)  50 (28.6)  90 (51.4) | 313 (16.8)  579 (31.0)  973 (52.2) | 1.21 (0.80 - 1.82)  0.93 (0.65 (1.34)  reference | 1.17 (0.71 - 1.93)  0.93 (0.63 - 1.36)  Reference |
| **Marital status :** Living alone | 13 (7.4) | 95 (5.1) | 1.50 (0.82 - 2.73) | 1.07 (0.55 - 2.08) |
| **Born in Canada** | 118 (67.4) | 1250 (67.0) | 1.02 (0.73 - 1.42) | 0.89 (0.59 - 1.34) |
| **Maternal ethnicity -**  Caucasian^‡^ | 145 (82.9) | 1563 (83.8) | 0.93 (0.62 - 1.41) | 0.79 (0.49 - 1.28) |
| **Parity**      0      1      ≥2 | 121 (69.1)  40 (22.9)  14 (8.0) | 987 (52.9)  627 (33.6)  251 (13.5) | Reference  **0.52 (0.36 - 0.75)**  **0.46 (0.26 - 0.81)** | Reference  **0.51 (0.34 - 0.75)**  **0.43 (0.24 - 0.80)** |
| **Body mass index**^§^**,** (kg/m^2^), mean ±SD | 24.1±4.6 | 25.0±5.3 | **0.96 (0.93 - 0.99)** | 0.92 (0.65 - 1.30) |
| **Maternal lifestyle during pregnancy** |  |  |  |  |
| Smoking | 41 (23.4) | 237 (12.7) | **2.10 (1.44 - 3.06)** | **2.01 (1.33 - 3.04)** |
| Coffee intake | 117 (66.9) | 1244 (66.7) | 1.01 (0.73 - 1.40) | 0.92 (0.65 - 1.30) |
| Illicit drug | 9 (5.1) | 59 (3.2) | 1.66 (0.81 - 3.41) | 1.17 (0.54 - 2.50) |
| **Use of Assisted reproductive technologies** | 18 (10.3) | 219 (11.7) | 0.86 (0.52 - 1.43) | 0.73 (0.43 - 1.26) |
| **Maternal comorbidities during pregnancy** |  |  |  |  |
| Diabetes | 1 (0.6) | 11 (0.6) | 0.97 (0.12 - 7.55) | 1.42 (0.17 - 12.11) |
| Asthma | 22 (12.6) | 130 (7.0) | **1.92 (1.19 - 3.11)** | **1.78 (1.07 - 2.96)** |
| Thyroid disease | 12 (6.9) | 114 (6.1) | 1.13 (0.61 - 2.09) | 1.27 (0.67 - 2.41) |
| Anemia | 4 (2.3) | 56 (3.0) | 0.76 (0.27 - 2.11) | 0.87 (0.33 - 2.27) |
| Hypercholesterolemia | 1 (0.6) | 20 (1.1) | 0.53 (0.07 - 3.97) | 0.54 (0.07 - 4.17) |
| Hypertension | 3 (1.7) | 16 (0.9) | 2.02 (0.58 - 6.99) | 3.01 (0.78 - 11.58) |
| Hepatitis | 1 (0.6) | 14 (0.8) | 0.76 (0.10 - 5.81) | 0.79 (0.10 - 6.37) |
| Gastro-intestinal disease | 11 (6.3) | 116 (6.2) | 1.01 (0.53 - 1.92) | 1.02 (0.53 - 1.96) |
| Sexually transmitted disease^\|\|^ | 12 (6.9) | 80 (4.3) | 1.64 (0.88 - 3.08) | 1.33 (0.69 - 2.57) |
| **Maternal flu during pregnancy** | 51 (29.1) | 525 (28.2) | 1.05 (0.75 - 1.48) | 1.08 (0.76 - 1.54) |
| **Season year of delivery^¶^**       2011       2012       2013 | 50 (28.5)  89 (50.9)  36 (20.6) | 452 (24.2)  1032 (55.4)  379 (20.4) | Reference  0.78 (0.54 - 1.12)  0.86 (0.55 - 1.35) | Reference  0.79 (0.54 - 1.15)  0.90 (0.56 - 1.45) |
| **SGA, Small for gestational age, 10^th^ percentile; CAD, Canadian dollars; OR, odds ratio**  *6 values deleted due to missing on the outcome, **N = 2040.**  **†**Adjusted for maternal age, gestational age, education, annual income, marital status, born in Canada, ethnicity, parity, body mass index, maternal lifestyle during pregnancy (smoking, coffee, illicit drug), use of assisted reproductive technologies, maternal comorbidities during pregnancy (diabetes, asthma, thyroid disease, anemia, hypercholesterolemia, hypertension, hepatitis, gastro-intestinal disease, sexually transmitted disease, maternal flu) and season year of delivery.  Depression is not considered due to null value in the low birth weight group.  ‡Caucasian= White, East-Asian, South-Asian, Arab/Occidental Asian.  ^§^BMI, 1^st^ visit Body Mass Index.  ^\|\|^Gonorrhea, Chlamydia, Condyloma, herpes  ^¶^From October 1^st^, 2010 to September 30^th^, 2011 season year = 2011; from October 1^st^ 2011 to September 30^th^, 2012 season year = 2012; from October 1^st^ 2012 to September 30^th^ 2013, season year = 2013. | | | | |

**Table S9. Unadjusted and adjusted association between in-utero patterns of exposure to flu vaccine and low birth weight**

| Characteristics | Low birth weight  N = 80 (%) | Normal birth weight  N = 1966 (%) | Unadjusted  OR  (95 % CI) | Adjusted*   OR (95 % CI) |
| --- | --- | --- | --- | --- |
| **Vaccine exposure patterns**        No exposure        Exposure 6 months before LMP        Exposure during 1^st^ trimester        Exposure during 2^nd^ trimester        Exposure during 3^rd^ trimester | 66 (82.5)  5 (6.3)  6 (7.5)  3 (3.7)  0 (0.0) | 1695 (86.2)  49 (2.5)  99 (5.0)  98 (5.0)  25 (1.3) | Reference  **2.62 (1.01 - 6.79)**  1.56 (0.66 - 3.68)  0.79 (0.24 - 2.55)  **-** | Reference  0.58 (0.09 - 3.94)  1.10  (0.23 - 5.29)  1.30 (0.27 - 6.17)  - |
| **Baby’s sex –** male | 50 (62.5) | 698 (49.2) | **1.72 (1.08 - 2.73)** | **3.00 (1.49 - 6.05)** |
| **Maternal age at delivery,** years, mean ±SD | 31.8±4.7 | 31.5±4.5 | 1.02 (0.97 - 1.07) | 1.03 (0.95 - 1.11) |
| **Gestational age at delivery, weeks,** mean±SD | 34.5±3.8 | 39.3±1.4 | **0.31 (0.26 - 0.38)** | **0.28 (0.23 - 0.35)** |
| **Post-secondary education** | 67 (83.8) | 1766 (89.8) | 0.58 (0.32 - 1.08) | 0.58 (0.21 - 1.57) |
| **Annual household income, CAD**      < 40 000      40 000 – 80 000      ≥ 80 000 | 12 (15.0)  30 (37.5)  38 (47.5) | 337 (17.1)  601 (30.6)  1028 (52.3) | 0.96 (0.50 - 1.87)  1.35 (0.83 - 2.20)  reference | 1.12 (0.40 - 3.12)  1.08 (0.51 - 2.30)  Reference |
| **Marital status :** Living alone | 7 (8.8) | 101 (5.1) | 1.77 (0.80 - 3.94) | 1.13 (0.30 - 4.26) |
| **Born in Canada** | 52 (65.0) | 1320 (67.1) | 0.91 (0.57 - 1.45) | 1.42 (0.61 - 3.30) |
| **Maternal ethnicity -**  Caucasian^†^ | 65 (81.3) | 1646 (83.7) | 0.84 (0.47 - 1.50) | 0.95 (0.35 - 2.55) |
| **Parity**      0      1      ≥2 | 48 (60.0)  23 (28.8)  9 (11.2) | 1062 (54.0)  648 (33.0)  256 (13.0) | Reference  0.79 (0.47 - 1.30)  0.78 (0.38 - 1.61) | Reference  0.51 (0.23 - 1.11)  0.61 (0.21 - 1.78) |
| **Body mass index^‡^,** (kg/m^2^), mean ±SD | 24.7±5.6 | 24.9±5.3 | 0.99 (0.85 - 1.03) | **0.93 (0.88 - 0.99)** |
| **Maternal lifestyle during pregnancy** |  |  |  |  |
| Smoking | 16 (20.0) | 263 (13.4) | 1.62 (0.92 - 2.84) | 1.48 (0.62 - 3.53) |
| Coffee intake | 50 (63.3) | 1316 (66.9) | 0.82 (0.52 - 1.31) | 0.86 (0.443 - 1.72) |
| Illicit drug | 2 (2.5) | 66 (3.4) | 0.74 (0.18 - 3.07) | 0.39 (0.04 - 4.44) |
| **Use of Assisted reproductive technologies** | 13 (16.3) | 224 (11.4) | 1.51 (0.82 - 2.78) | 0.83 (0.31 - 2.21) |
| **Maternal comorbidities during pregnancy** | | | | |
| Diabetes | 2 (2.5) | 10 (0.5) | 5.02 (1.08 - 23.28) | 2.11 (0.11 - 40.04) |
| Asthma | 8 (10.0) | 145 (7.4) | 1.40 (0.66 - 2.95) | 1.72 (0.59 - 5.12) |
| Thyroid disease | 8 (10.0) | 119 (6.1) | 1.73 (0.81 - 3.66) | 1.94 (0.59 - 6.38) |
| Anemia | 2 (2.5) | 60 (3.1) | 0.82 (0.20 - 3.39) | 0.24 (0.02 -3.14) |
| Hypertension | 3 (3.8) | 16 (0.8) | 4.75 (1.36 - 16.64) | 1.09 (0.12 - 10.16) |
| Hepatitis | 2 (2.5) | 13 (0.7) | 3.85 (0.86 - 17.37) | 4.50 (0.28 - 72.03) |
| Gastro-intestinal disease | 3 (3.8) | 125 (6.4) | 0.57 (0.18 - 1.84) | 0.22 (0.03 - 1.47) |
| Sexually transmitted disease^§^ | 2 (2.5) | 90 (4.6) | 0.53 (0.13 - 2.21) | 0.52 (0.10 - 2.82) |
| Depression | 1 (1.3) | 14 (0.7) | 1.77 (0.23 - 13.59) | 2.19 (0.11 - 43.14) |
| **Maternal flu during pregnancy** | 23 (28.8) | 554 (28.2) | 1.03 (0.63 - 1.69) | 0.86 (0.41 - 1.81) |
| **Season year of delivery**^\|\|^       2011       2012       2013 | 21 (26.3)  48 (60.0)  11 (13.7) | 482 (24.5)  1079 (54.9)  405 (20.6) | Reference  1.02 (0.61 - 1.72)  0.62 (0.30 - 1.31) | Reference  1.13 (0.50 - 2.56)  0.82 (0.27 - 2.49) |
| **SD, standard deviation; CAD, Canadian dollars; OR, odd ratio**  *****Adjusted for maternal age, gestational age, education, annual income, marital status, born in Canada, ethnicity, parity, body mass index, maternal lifestyle during pregnancy (smoking, coffee, illicit drug), use of assisted reproductive technologies, maternal comorbidities during pregnancy (diabetes, asthma, thyroid disease, anemia, hypertension, hepatitis, gastro-intestinal disease, sexually transmitted disease, depression and maternal flu)  and season year of delivery.  Hypercholesterolemia is not considered due to null value in the low birth weight group.  †Caucasian= White, East-Asian, South-Asian, Arab/Occidental Asian.  ^‡^BMI, 1^st^ visit Body Mass Index.  ^§^Gonorrhea, Chlamydia, Condyloma, herpes  ^\|\|^From October 1^st^, 2010 to September 30^th^, 2011 season year = 2011; from October 1^st^ 2011 to September 30^th^, 2012 season year = 2012; from October 1^st^ 2012 to September 30^th^ 2013, season year = 2013. | | | | |

## Table S10. Unadjusted and adjusted association between in-utero patterns of exposure to flu vaccine and all cause hospitalization*.

| Characteristics | Hospitalization  N = 190 (%) | No hospitalization  N = 1451 (%) | Unadjusted  OR  (95 % CI) | Adjusted^†^   OR (95 % CI) |
| --- | --- | --- | --- | --- |
| **Vaccine exposure patterns**        No exposure        Exposure 6 months before LMP        Exposure during 1^st^ trimester        Exposure during 2^nd^ trimester        Exposure during 3^rd^ trimester | 162 (85.3)  6 (3.2)  12 (6.3)  3 (4.2)  2 (1.0) | 1246 (85.9)  42 (2.9)  72 (5.0)  74 (5.1)  17 (1.0) | Reference  1.10 (0.46 - 2.63)  1.28 (0.68 - 2.41)  0.83 (0.39 - 1.76)  0.91 (0.21 - 3.95) | Reference  0.94 (0.38 - 2.35)  1.41 (0.71 - 2.79)  0.80 (0.37 - 1.74)  0.84 (0.19 - 3.76) |
| **Baby’s sex –** male | 81 (42.6) | 735 (50.7) | **0.72 (0.53 - 0.98)** | **0.73 (0.52 - 0.99)** |
| **Maternal age at delivery,** years, mean ±SD | 31.8±4.3 | 31.4±4.4 | 1.02 (0.99 - 1.06) | 1.01 (0.97 - 1.05) |
| **Gestational age  at delivery, weeks,** mean ±SD | 38.6±2.2 | 39.2±1.6 | **0.83 (0.77 - 0.90)** | **0.82 (0.75 - 0.90)** |
| **Post-secondary education** | 178 (93.7) | 1318 (90.8) | 1.50 (0.81 - 2.76) | 1.58 (0.74 - 3.08) |
| **Annual household income, CAD**      < 40 000      40 000 – 80 000      ≥ 80 000 | 28 (14.7)  63 (33.2)  99 (52.1) | 218 (15.0)  429 (29.6)  804 (55.4) | 1.04 (0.67 - 1.63)  1.19 (0.85 - 1.67)  reference | 1.34 (0.78 - 2.26)  1.28 (0.77 - 1.85)  Reference |
| **Marital status :** Living alone | 6 (3.2) | 73 (5.0) | 0.62 (0.26 - 1.44) | 0.55 (0.22 - 1.39) |
| **Born in Canada** | 131 (69.0) | 1023 (70.5) | 0.93 (0.67 - 1.29) | 1.03 (0.68 - 1.56) |
| **Maternal ethnicity -**  Caucasian^‡^ | 161 (84.7) | 1244 (85.7) | 0.92 (0.61 - 1.41) | 1.06 (0.64 - 1.74) |
| **Parity**      0      1      ≥2 | 86 (45.3)  79 (41.6)  25 (13.1) | 831 (57.3)  454 (31.3)  166 (11.4) | Reference  **1.68 (1.21 - 2.33)**  1.46 (0.81 - 2.34) | Reference  **1.57 (1.11 - 2.23)**  1.30 (0.77 - 2.19) |
| **Body mass index^§^,** (kg/m^2^), mean ±SD | 25.2±4.9 | 24.9±5.2 | 1.01 (0.99 - 1.04) | 1.00 (0.97 - 1.03) |
| **Maternal lifestyle during pregnancy** | | | | |
| Smoking | 18 (9.5) | 201 (13.9) | 0.65 (0.39 - 1.08) | 0.70 (0.41 - 1.20) |
| Coffee intake | 127 (66.8) | 983 (67.8) | 0.96 (0.70 - 1.32) | 1.06 (0.77 - 1.49) |
| Illicit drug | 5 (2.6) | 47 (3.2) | 0.81 (0.32 - 2.06) | 0.95 (0.36 - 2.54) |
| **Use of Assisted reproductive technologies** | 16 (8.4) | 173 (11.9) | 0.68 (0.40 - 1.16) | 0.62 (0.35 - 1.11) |
| **Maternal comorbidities during pregnancy** | | | | |
| Diabetes | 3 (1.6) | 6 (0.4) | 3.86 (0.96 - 15.58) | 3.06 (0.68 - 13.88) |
| Asthma | 16 (8.4) | 111 (7.7) | 1.10 (0.64 - 1.92) | 1.29 (0.73 - 2.28) |
| Thyroid disease | 13 (6.8) | 92 (6.3) | 1.09 (0.60 - 1.98) | 1.20 (0.64 - 2.26) |
| Anemia | 2 (1.1) | 37 (2.6) | 0.41 (0.10 - 1.70) | 0.31 (0.08 - 1.33) |
| Hypertension | 1 (0.5) | 14 (1.0) | 0.54 (0.07 - 4.15) | 0.24 (0.03 - 2.11) |
| Hepatitis | 4 (2.1) | 7 (0.5) | **4.44 (1.29 - 15.30)** | **4.72 (1.28 - 17.37)** |
| Gastro-intestinal disease | 15 (7.9) | 87 (6.0) | 1.34 (0.76 - 2.38) | 1.42 (0.78 - 2.58) |
| Sexually transmitted disease^\|\|^ | 11 (5.8) | 68 (4.7) | 1.25 (0.65 - 2.41) | 1.34 (0.67 - 2.66) |
| **Maternal flu during pregnancy** | 58 (30.5) | 418 (28.8) | 1.09 (0.78 - 1.51) | 1.01 (0.72 - 1.43) |
| **Season year of delivery^¶^**       2011       2012       2013 | 39 (20.5)  102 (53.7)  49 (25.8) | 310 (21.4)  851 (58.6)  290 (20.0) | Reference  0.95 (0.64 - 1.41)  1.34 (0.86 - 2.11) | Reference  0.97 (0.65 - 1.46)  1.51 (0.93 - 2.43) |
| SD, standard deviation; CAD, Canadian dollars; OR, odd ratio  *405 values deleted due to missing on the outcome, N = 1641.  †Adjusted for maternal age, gestational age, education, annual income, marital status, born in Canada, ethnicity, parity, body mass index, maternal lifestyle during pregnancy (smoking, coffee, illicit drug), use of assisted reproductive technologies, maternal comorbidities during pregnancy (diabetes, asthma, thyroid disease, anemia, hypertension, hepatitis, gastro-intestinal disease, sexually transmitted disease, maternal flu) and season year of delivery. Depression and Hypercholesterolemia are not considered due to null value in the hospitalization group.  ‡Caucasian= White, East-Asian, South-Asian, Arab/Occidental Asian.  §BMI, 1st visit Body Mass Index.  \|\|Gonorrhea, Chlamydia, Condyloma, herpes  -From October 1st, 2010 to September 30th, 2011 season year = 2011; from October 1st 2011 to September 30th, 2012 season year = 2012; from October 1st 2012 to September 30th 2013, season year = 2013. | | | | |

Table S11. Summary of different opinions from past findings to the present status of thought regarding the recommendation of vaccination for pregnant persons

| Study | Key Findings |
| --- | --- |
| Munoz, 2012 | Influenza vaccine has been administered to pregnant persons in the United States for over six decades. The Centers for Disease Control and Prevention and the ACOG recommended influenza vaccination for all pregnant persons since 2004. |
| Canada public guidelines on influenza vaccination | In Canada, prior to 2007, influenza vaccination during pregnancy was encouraged, particularly among individuals at increased risk of influenza-related complications |
| Canadian Immunization Guide Chapter | In 2007, the National Advisory Committee on Immunization (NACI) in Canada, recommended annual seasonal non-live influenza vaccination in pregnant individuals, and in children aged 6 months and older |
| Getahun et al., Nunes et al., Thompson et al. | In these studies, Influenza vaccine was associated with a decreased risk of maternal influenza. Thus, recommending the vaccine during pregnancy |
| Donahue et al. | In this study, the authors showed that influenza vaccine was associated with an increased risk of spontaneous abortion. |
| Donahue et al. | The same authors of the previous study, found that influenza vaccine is not associated with an increased risk of spontaneous abortion, using a different period of influenza season. |
| Steinhoff et al. | These authors observed that prenatal influenza immunization might reduce birth outcomes |
| Giles et al. | The authors showed that influenza vaccine reduced the risk of preterm birth and low birthweight, but was not significantly associated with SGA |
| Rolfes et al. | Prenatal influenza vaccine was associated with a significant decrease of the risk of preterm birth |
| Nunes et al. | Prenatal influenza vaccine was associated with a significant decrease of low birthweight, but was not significantly associated with SGA |
| Fell et al., Jeong et al., Omer et al. | In these studies, the authors showed that prenatal influenza vaccine was not associated with PTB, LBW or SGA |
